# Macrophages orchestrate breast cancer early dissemination and metastasis

**DOI:** 10.1038/s41467-017-02481-5

**Published:** 2018-01-02

**Authors:** Nina Linde, Maria Casanova-Acebes, Maria Soledad Sosa, Arthur Mortha, Adeeb Rahman, Eduardo Farias, Kathryn Harper, Ethan Tardio, Ivan Reyes Torres, Joan Jones, John Condeelis, Miriam Merad, Julio A. Aguirre-Ghiso

**Affiliations:** 1 Division of Hematology and Oncology, Department of Medicine, Tisch Cancer Institute, Black Family Stem Cell Institute, Icahn School of Medicine at Mount Sinai, New York, NY 10029 USA; 2 Department of Otolaryngology, Tisch Cancer Institute, Black Family Stem Cell Institute, Icahn School of Medicine at Mount Sinai, New York, NY 10029 USA; 3 Department of Oncological Sciences, The Immunology Institute, Tisch Cancer Institute, Icahn School of Medicine at Mount Sinai, New York, NY 10029 USA; 4 Human Immune Monitoring Core, Icahn School of Medicine at Mount Sinai, New York, NY 10029 USA; 5 Department of Anatomy and Structural Biology, Integrated Imaging Program, Gruss Lipper Biophotonics Center, Albert Einstein College of Medicine, 1300 Morris Park Avenue, Bronx, NY 10461 USA; 6 Present Address: Merck KGaA, Frankfurter Str. 250, Postcode: A025/301, Darmstadt, 64293 Germany; 7 Present Address: Department of Pharmacological Sciences, Icahn School of Medicine at Mount Sinai, New York, NY 10029 USA; 8 Department of Immunology, University of Toronto, Toronto, ON M5S 1A8 USA

## Abstract

Cancer cell dissemination during very early stages of breast cancer proceeds through poorly understood mechanisms. Here we show, in a mouse model of HER2^+^ breast cancer, that a previously described sub-population of early-evolved cancer cells requires macrophages for early dissemination. Depletion of macrophages specifically during pre-malignant stages reduces early dissemination and also results in reduced metastatic burden at end stages of cancer progression. Mechanistically, we show that, in pre-malignant lesions, CCL2 produced by cancer cells and myeloid cells attracts CD206^+^/Tie2^+^ macrophages and induces Wnt-1 upregulation that in turn downregulates E-cadherin junctions in the HER2^+^ early cancer cells. We also observe macrophage-containing tumor microenvironments of metastasis structures in the pre-malignant lesions that can operate as portals for intravasation. These data support a causal role for macrophages in early dissemination that affects long-term metastasis development much later in cancer progression. A pilot analysis on human specimens revealed intra-epithelial macrophages and loss of E-cadherin junctions in ductal carcinoma in situ, supporting a potential clinical relevance.

## Abstract (web summary)

Early dissemination of cancer cells has been reported to occur in certain breast cancer models. Here the authors show that intra-epithelial macrophages in the early pre-cancer lesions drive early cancer cell dissemination through Wnt-1 secretion and that such events impact the later development of metastasis.

## Introduction

The paradigm of cancer metastasis states that dissemination and metastasis occur when advanced aggressive tumors acquire invasive mechanisms. The finding that dissemination does not only occur from evolutionary late-stage invasive tumors has challenged the uniqueness of this model^1^. Large cohort patient studies^2–5^ and studies with spontaneous mouse tumor models^6^ showed that dissemination also occurs during early stages of cancer when lesions are diagnosed by light microscopy as pre-malignant or pre-invasive. In addition, cancer of unknown primary is a relatively frequent event in solid cancers where metastases develop without the presence of an obvious primary tumor mass that evolved to become invasive^7^.

The “early dissemination” definition was refined by Husemann et al.^6^ when they showed that early disseminated cancer cells (DCCs) originate at times when lesions are only defined in situ by light microscopy (e.g., ductal carcinoma in situ (DCIS) in humans and mammary intra-epithelial neoplasia in mice), but dissemination occurs and early DCCs show few genetic aberrations. Following previous work^6^, we found that in MMTV-HER2 early lesions there is a sub-population of HER2^+^/P-p38^lo^/P-ATF2^lo^/TWIST^hi^/E-cadherin^lo^ disseminating cancer cells that reach distant organs and initiate metastasis^8, 9^. Our studies revealed that HER2^+^ early cancer cells deregulate mechanisms of motility and invasion activated during mammary tissue branching morphogenesis^8, 9^. The remarkable finding was that early DCCs are endowed with latent metastasis-initiating capacity^8, 9^. Women treated for DCIS can develop metastases without ever developing any subsequent local invasive breast cancer^10–14^. This might indicate that, albeit at low frequency, early DCCs can unpredictably form metastases in patients. Early dissemination is not a rarity of breast cancer models (MMTV-HER2 and MMTV-PyMT models^6, 8, 9^), as it also occurs in spontaneous mouse models of melanoma^15^ and pancreatic cancer^16^.

The mechanistic analysis of early dissemination has been primarily early cancer cell-centric^8, 9^. Since early DCCs displayed fewer genetic alterations than the late DCC counterparts^4, 6, 8^, and the mechanism of dissemination resembled steps of mammary morphogenesis^8, 9^, we hypothesized that early dissemination might be driven by micro-environmental mechanisms that control epithelial cell motility and invasion during mammary tissue development^17, 18^.

The mammary epithelium forms post-natally during adolescence in a process called branching morphogenesis where rapidly dividing epithelial cells elongate the terminal end bud into the fat pad and bifurcate into the ductal tree. Macrophages are key regulators of branching morphogenesis during mammary gland development^19, 20^, arguing that normal mammary epithelial cells cooperate with these innate immune cells for invasive processes. These data led to the discovery of macrophages as powerful drivers of intravasation from invasive breast cancer tumors via the establishment of tumor microenvironments of metastasis (TMEM)^21^. This follows a streaming process where breast cancer cells recruit macrophages through colony-stimulating factor 1 (CSF1) production and then cancer cell motility is stimulated via macrophage-derived epidermal growth factor (EGF)^22^. Additionally, macrophages can induce an epithelial-to-mesenchymal transition (EMT) in malignant cells^23, 24^. Elegant studies by Pollard and colleagues^25^ ^[,26^ have also shown that macrophages play key roles in the maintenance of lung metastasis. However, the role of macrophages in the process of dissemination during evolutionary early stages of breast cancer progression remained unexplored.

Here we show that the branching morphogenesis program is altered by oncogenes early in cancer development. CD206^hi^ macrophages in the mammary tissue are attracted by early cancer cells from the stroma into the epithelial layer of lesions defined as mammary intra-epithelial neoplasia in mice (similar to DCIS in humans)^27^. In the MMTV-HER2 model this process depends on HER2-NF-κB-mediated induction of CCL2. Our data suggest that intra-epithelial macrophages respond to CCL2, which in turn can stimulate macrophages to produce Wnt-1, leading to disruption of E-cadherin junctions between early cancer cells. Before tumors form, these events result in early dissemination microenvironments that propel active intravasation and dissemination to the lung, which was efficiently blocked by macrophage depletion. Transient depletion of macrophages in mice before the formation of invasive tumors reduced lung metastatic burden later in mice life. Our results suggest a previously unrecognized role for local attraction of macrophages from the stroma into the ductal epithelium to favor dissemination of cancer cells much earlier than growth is induced by the oncogene. Our work also reveals a role for early DCCs in supporting late metastasis development, which is agreement with our recent studies supporting that ~79% of metastases in the MMTV-HER2 model descend from early DCCs.

## Results

### Macrophages infiltrate Her2^+^ early lesions

**Fig. 1:**
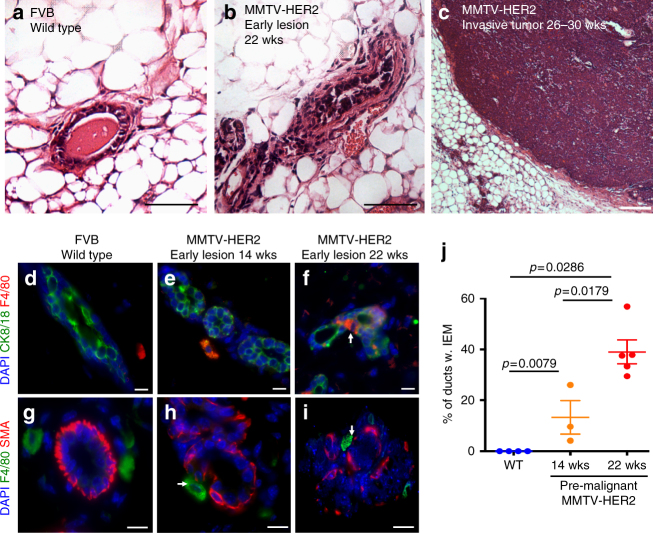
Macrophages enter the ductal epithelial layer in early breast cancer lesions. H&E staining of mammary gland sections show progression from healthy mammary ducts in FVB wild-type glands (age 20wks; **a**) to early lesions classified as hyperplasia and mammary intra-epithelial neoplasia (age 22wks; **b**) to invasive tumors (age 26–30wks; **c**) in the MMTV-HER2 mouse model. Bars: 100 μm. Mammary glands from FVB wild-type (20wks; **d**) or pre-malignant MMTV-HER2 mice at age 14wks (**e**) and 22wks (**f**) were stained against F4/80 (macrophages) and CK8/18 (epithelial cells) and against F4/80 and smooth muscle actin (SMA) (**g–i**). Bars: 10 μm. The mean±SEM of the percentage of ducts containing IEM is shown; FVB: *N* = 4 mice, 14wks; *N* = 3 mice, 22wks; *N* = 5 mice (**j**). *P* values were calculated with 95% confidence by Mann–Whitney test

We asked whether the HER2 oncogene might attract macrophages to orchestrate early dissemination. We used MMTV-HER2 mice as a murine model of breast cancer since these show slow progression from early lesions such as hyperplasia and mammary intra-epithelial neoplasia (Fig. 1a, b, Supplementary Table 1), the latter a similar lesion to DCIS^27^, to invasive tumors (Fig. 1c, Supplementary Table 1). We stained MMTV-HER2 mammary gland sections for the murine macrophage marker F4/80 before we could detect any signs of invasive tumor masses in serial sections of mammary tissue or even enhanced proliferation in HER2^+^early lesions^28^. Macrophages were located to the stroma outside of mammary ducts in healthy FVB wild-type (WT) animals (Fig. 1d, Supplementary Table 1). This was also true in young 14-week-old MMTV-HER2 mice (Fig. 1e, Supplementary Table 1). However, when MMTV-HER2 mice progressed over time but still showed no signs of tumor masses or enhanced proliferation^28^, macrophages were often localized inside the luminal epithelial layer of early lesions as demonstrated by co-staining of F4/80 and cytokeratin 8/18 (CK8/18) (Fig. 1f, Supplementary Table 1). We hypothesized that as macrophages enter the early lesions, they might disrupt the architecture of the duct. Close inspection of sections co-stained for α-smooth muscle actin and F4/80 showed that the myoepithelial cell layer was frequently disrupted at sites where macrophages were in immediate contact with the duct (Fig. 1g–i, Supplementary Table 1). Quantification of the abundance of intra-epithelial macrophages confirmed that the incidence of ducts with intra-epithelial macrophages correlated with HER2 upregulation and disease progression (Fig. 1j, Supplementary Table 1).

### Macrophages dismantle E-cadherin junctions in early lesions

**Fig. 2:**
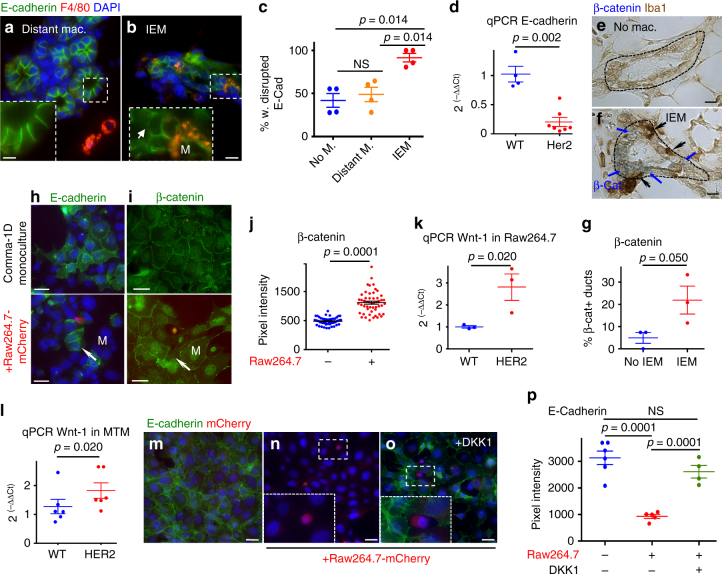
Intra-epithelial macrophages (IEMs) induce an EMT-like response in early cancer cells. Twenty-week-old MMTV-HER2 mouse mammary glands were stained against E-cadherin and F4/80. E-cadherin localization was analyzed dependent on whether macrophages did not make direct contact to the duct (no M. or distant M.; **a**) or whether ducts contain IEMs (**b**). The percentage of individual epithelial cells with disrupted E-cadherin was quantified in four mice and is shown as mean±SEM (**c**). Statistical analysis: Mann–Whitney test. E-cadherin mRNA expression in whole mammary glands of FVB wild-type (WT, 20wks, *N* = 4) or 20-week-old MMTV-HER2 (*N* = 7) mice was determined by qPCR and is shown as mean±SEM by Mann–Whitney test. **d** Twenty-four-week-old MMTV-HER2 mammary glands (*N* = 3 per group) were stained against β-catenin and Iba1, a macrophage marker. **e**, **f** β-Catenin^+^ early cancer cells (blue) were more frequent in ducts containing IEMs. **g** Plots shown as mean±SEM by Mann–Whitney test. The mammary epithelial cell line Comma-1D was grown as a monolayer before the addition of mCherry expressing Raw264.7 macrophages. E-cadherin (**h**) or β-catenin (**i**) was stained and the intra-nuclear signal intensity of β-catenin was quantified (**j**). Plots show nuclear β-catenin signal intensity in individual cells; independent experiments *N* = 3, Student’s *t* test. **k**, **l** Conditioned medium was harvested from primary mammospheres of 20–22-week-old WT or pre-malignant MMTV-HER2 mice and added to Raw264.7 macrophages or mammary tissue macrophages (MTMs) isolated from pre-malignant MMTV-HER2 mammary glands. Wnt-1 expression is depicted as mean±SEM of three technical replicates. Statistical significance was determined by Student’s *t* test with 95% confidence interval; individual experiments *N* = 3 for Raw264.7 and *N* = 2 for MTMs. Comma-1D cells were grown as monolayers (**m**) and Raw264.7-mCherry macrophages were added (**n**) and additionally treated with DKK1 (**o**). E-cadherin signal intensity in whole section was quantified and is shown as mean±SEM, where each dot represents one microscopic field; independent experiments *N* = 2, Student’s *t* test. The pixel intensity of the cells in (**n**) is background pixel signal for the green channel, ~1000 in pixel intensity

We hypothesized that HER2 might aberrantly activate a mechanism of invasion and motility involving macrophages in early lesions. We found that intra-epithelial macrophages were associated with a strong local downregulation of E-cadherin junctions *in vivo* in early lesions cells located directly adjacent to macrophages (1–2 cell diameter away) (Fig. 2a–c, Supplementary Table 2). This was paralleled by a general downregulation of E-cadherin mRNA in early lesions compared to WT glands (Fig. 2d, Supplementary Table 2), which had been reported previously^9^. Additionally, β-catenin levels (blue signal Fig. 2e–g) were increased in early lesions containing intra-epithelial macrophages as measured by dual-color immunohistochemistry (IHC) (Fig. 2e–g, Supplementary Table 2). A loss of E-cadherin and translocation of β-catenin to the nucleus could also be induced in vitro when Raw264.7 macrophages were added to Comma-1D healthy mammary epithelial cell monolayers used as readout for epithelial junction formation (Fig. 2h–j, m, n, p). The loss of E-cadherin junctions in epithelial cells adjacent to macrophages suggested that macrophages might produce cues that stimulate a loss of E-cadherin junctions as observed during the EMT. Macrophages can produce Wnt ligands^29–31^ which are potent inducers of an EMT. We therefore tested the response of Raw264.7 macrophages or primary mammary tissue macrophages isolated from early lesions to conditioned media from healthy epithelial cells or from HER2^+^ early cancer cells. Only conditioned media derived from HER2^+^ cells induced an upregulation of Wnt-1 (Fig. 2k, l, Supplementary Table 2); no changes were detected for Wnt-3a upon treatment with the HER2^+^ cells conditioned media and Wnt-5a and Wnt-7 were not detectable in either mammary tissue macrophages or Raw264.7 cells (Supplementary Fig. 1G). Our published data also showed that Wnt-1 is not produced by the early lesions^8, 9^. Comma-1D cells also do not produce Wnt-1^32^, arguing that macrophages are the main source of Wnt-1. However, HER2^+^ early cancer cells do produce other Wnt ligands^8, 9^ that could further enhance a Wnt signaling pathway. The loss of E-cadherin junctions in Comma-1D cells induced by Raw264.7 macrophages was reversed by the addition of DKK1, an inhibitor of canonical Wnt signaling, to the co-cultures (Fig. 2m–p). Further, Comma-1D cells exposed to conditioned media from Raw264.7 cells immuno-depleted via precipitation from Wnt-1 with a specific antibody (Supplementary Fig. 1A, B) were able to restore E-cadherin junctions, when compared to immunogobulin G (IgG)-depleted conditioned media that displayed marginal E-cadherin junction formation (Supplementary Fig. 1A, B). Our data support that downregulation of *Cdh1* mRNA junctions and β-catenin nuclear translocation in early lesions cells results from HER2-dependent attraction of Wnt-1-secreting macrophages from the stroma into the early lesions. Because HER2^+^ early cancer cells also produce other Wnt ligands, we further conclude that the downregulation of E-cadherin junctions may be due to the combined effect of early cancer cell-derived and macrophage-derived Wnt ligands.

Macrophages that produce Wnt ligands^31^ are known to associate with tumor cells that interact with the TMEM structures. A TMEM structure is composed of a macrophage, a MENA^hi^ tumor cell and endothelial cells and serve as the portal for intravasation^21, 33^. The presence of macrophages in early lesions prompted us to test if TMEMs are formed in these HER2 early lesions as found in the PyMT model early lesions^21^. Using a clinically validated test^33^ it was shown that early cancer lesions in the MMTV-HER2 and MMTV-PyMT^21^ models were efficiently forming TMEM (Supplementary Fig. 1C, D). Our data (Supplementary Fig. 1C, D, Supplementary Table 7) reveals that the HER2^+^ model also shows formation of TMEM structures which can operate as portals for intravasation^21, 33^. These TMEM structures occur in approximately 10% of ducts at 17 weeks in MMTV-HER2 animals (Supplementary Fig. 1E, Supplementary Table 7). This frequency is in range with the frequency of intra-epithelial macrophages in pre-malignant lesions in Fig. 1j. Importantly, compared to WT mammary tissue, early lesions in the MMTV-PyMT model also showed a significant (*p* ≤ 0.0001, Mann–Whitney test) downregulation of E-cadherin (Supplementary Fig. 2A, Supplementary Table 7), arguing that PyMT-initiated signaling might converge on similar signals as HER2 to attract macrophages from the stroma into the early lesions and downregulate E-cadherin junctions. As reported^21^, normal mammary tissue in FvB mice was negative for TMEM (Supplementary Fig. 1F).

### Macrophage depletion prevents early dissemination

**Fig. 3:**
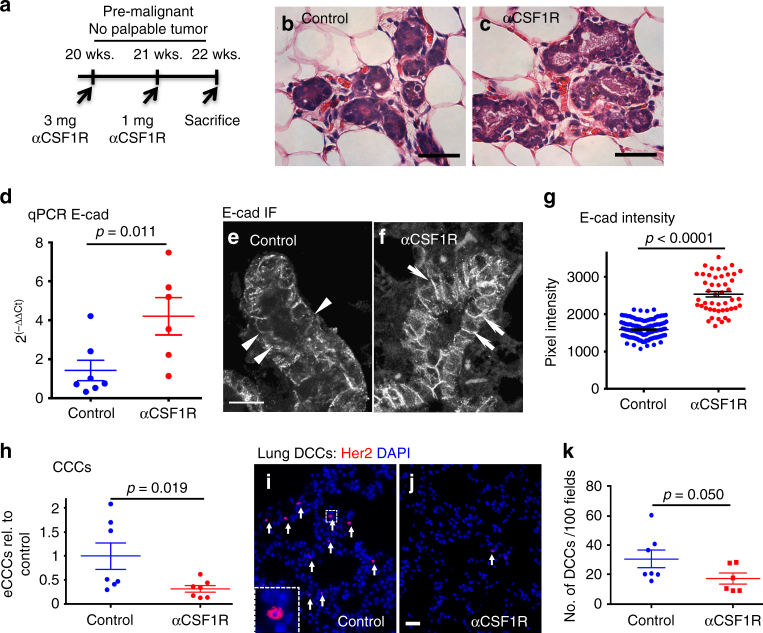
Macrophage depletion during pre-malignant stages prevents early cancer cell dissemination. Twenty-week-old pre-malignant MMTV-HER2 mice were treated with the anti-CSF1R ASF98 antibody and animals were harvested after 2 weeks with no signs of invasive carcinoma (**a**). Analysis of H&E staining of mammary gland sections confirmed the absence of invasive lesions (**b**, **c**; bars: 100 μm). E-cadherin expression in whole mammary glands was determined (**d**; mean±SEM; control 7, anti-CSF1R 6 animals; statistical analysis: Mann–Whitney test) and by immunofluorescent staining against E-cadherin in mammary gland sections (**e**,** f**; bars: 10 μm). E-cadherin signal intensity was measured in individual regions of cell junctions in three animals per group (**g**). *P* values were calculated with 95% confidence interval by Mann–Whitney test. Early circulating cancer cells (eCCCs) were quantified in mice per group as the amount of HER2 and CK8/18^+^ eCCs/ml peripheral blood. Values were normalized to the mean of controls and are shown as mean±SEM (**h**) depicts normalized mean±SEM; seven animals per group; statistical analysis: Mann–Whitney test. Disseminated HER2^+^ eCCs were quantified in lung sections (**i**, **j**; bars: 25 μM) and were quantified as the average of HER2^+^ cells per 100 randomly chosen microscopic fields (**k**). Mean±SEM are shown; statistical analysis: Mann–Whitney test

We next tested whether macrophage mobilization into early lesions where they induce an EMT in early cancer cells leads to early dissemination. CSF1 receptor (CSF1R) is expressed by most tissue-resident macrophages and is required for macrophages' survival in tissues^35^. Thus, we injected MMTV-HER2 mice during early lesions with a blocking antibody to CSF1R to eliminate macrophages from early lesions or as controls with phosphate-buffered saline (PBS) or an isotype-matched IgG (Fig. 3a, Supplementary Fig. 2B, Supplementary Table 7). CSF1R blockade led to the depletion of tissue-resident CD11b^+^/F4/80^+^/Gr1^−^ macrophages compared to PBS-treated and IgG-treated animals (Supplementary Fig. 2B, C, Supplementary Table 7). Importantly, IgG control did not alter the relative or total levels of F4/80^+^CD11b^+^ macrophages, CD11b^+^F4/80^neg^ monocytes or F4/80^+^CD206^hi^ or F4/80^+^CD206^lo^ macrophages. Dendritic cell and neutrophil numbers and percentages from the CD45^+^ gate were also not affected by the IgG (Supplementary Fig. 2C, Supplementary Table 7). CSF1R-blocking antibody treatment did deplete significantly (*p*<0.05) the F4/80^+^CD206^hi^ macrophage population and monocytes, while neutrophils and dendritic cells remain unchanged (Supplementary Fig. 2C, Supplementary Table 7). Quantification of immunofluorescence (IF) staining images for F4/80 in HER2^+^ early lesions (Supplementary Fig. 2E, Supplementary Table 7) confirmed a significant reduction in the number of intra-epithelial macrophages when CSF1R was blocked compared to all controls (Supplementary Fig. 2B, C, and E; *p*<0.05; Supplementary Table 7). We confirmed that at the end of the experiment, no tumor masses had formed, by inspecting whole mounts of mammary glands (Supplementary Fig. 2G, H, Supplementary Table 7) and hematoxylin–eosin (H&E) staining of serially sectioned mammary tissue (Fig. 3b, c, Supplementary Table 3). Macrophage depletion was accompanied by a significant reduction in the number of hyperplastic ducts (Fig. 3b–g, Supplementary Table 3, and Supplementary Fig. 2F; *p*<0.05; Supplementary Table 7) and a tissue-wide upregulation of E-cadherin mRNA (Fig. 3d) and E-cadherin-based junctions compared to PBS (Fig. 3e–g, Supplementary Table 3) or IgG controls (Supplementary Fig. 2D, Supplementary Table 7). Overall, we conclude that macrophages contribute to the loss of E-cadherin mRNA and junctions and disrupted mammary tissue architecture in early lesions.

The above changes correlated with the finding that CSF1R blockade significantly (*p* = 0.019) reduced the number of early circulating cancer cells (Fig. 3h, Supplementary Table 3). Accordingly, CSF1R blockade also reduced early DCC burden in target organs as measured by the detection of the transgene surface HER2 expressing early DCCs in the lungs (Fig. 3i–k, Supplementary Table 3). To rule out that HER2^+^ cells might be macrophages that engulfed early cancer cells in the mammary tissue and migrated to the lung, we tested the fraction of cells in the lungs in MMTV-HER2 animals that might be double positive for HER2 and macrophage markers (F4/80) (Supplementary Fig. 3A, Supplementary Table 7). Only ~2 cells per field of view (FOV) were HER2/F4/80 double positive vs. a median of 46 DCCs/FOV were HER2^+^/F4/80^−^. This results in a frequency of 0.04 or 4% of all HER2^+^ cells being double positive. This argues that only 4% of HER2^+^ cells could be confused for a macrophage engulfing HER2^+^ cells in the lung or traveling to the lung with the engulfed cells. We conclude that macrophages play a critical role in the ability of early cancer cells to acquire an invasive and disseminating phenotype.

### Early dissemination macrophages fuel late metastasis

**Fig. 4:**
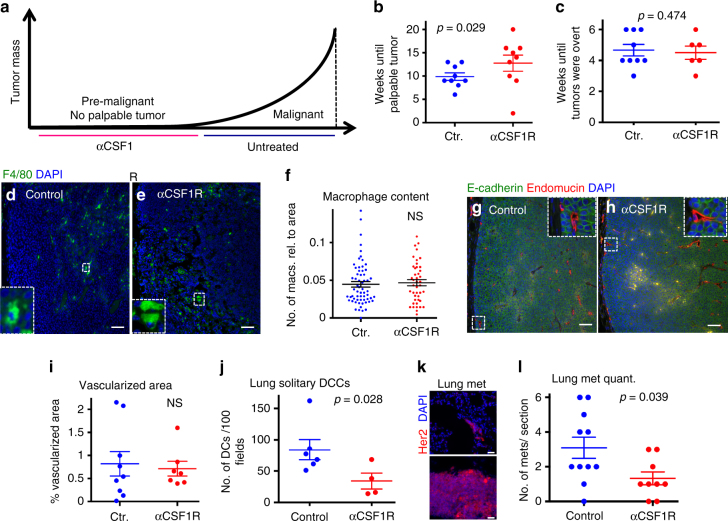
Early disseminated cancer cells contribute to metastasis formation. Macrophages were depleted from pre-malignant MMTV-HER2 mice by ASF98 treatment starting at week 18. Treatment was stopped when mice developed palpable tumors (1–3 mm average). **a** Mice were left until tumors reached 1 cm in diameter and then sacrificed. **b** Time from beginning of treatment at age wk18 until development of palpable tumors as mean±SEM (9 mice each, 23–38 wks old). **c** Time from formation of palpable tumors until tumors were overt (26-43 wks old) as mean±SEM (control *N* = 9, anti-CSF1R *N* = 6). Macrophages in sections of overt tumors (at least three animals per group) were identified by staining against F4/80 at the end of the experiment (**d**, **e**; bar: 100 μm; zoom factor in insets 5x) and quantified as the number of macrophages relative to tumor area (**f**; statistical analysis: Mann–Whitney test). Vascularization of overt tumors was analyzed by staining against endomucin, an endothelial cell marker (**g**, **h**; bar: 100 μm; zoom factor in insets 4x) and quantified as endomucin^+^ area/tumor in at least three animals combined (**i**; mean±SEM; statistical analysis: Mann–Whitney test). Solitary DCCs in lung sections and metastases defined as cell clusters bigger than three cells were quantified in lung sections stained against HER2 (**j**–**l**, bar: 25 μm). For solitary cell analysis, the average of DCCs or metastases per 100 fields was counted; each dot represents one lung section. For metastasis analysis, the total number of metastases per lung sections was quantified and plotted (**j**). Number of mice *N* = 6 (control) and *N* = 4 (αCSF1R) animals combined; statistical analysis: Mann–Whitney test

We next tested whether macrophage-regulated early dissemination contributed to metastasis formation. To this end, we blocked CSF1R only during early asymptomatic stages of cancer, starting at age week 18, and stopped as soon as tumors became palpable (size <3 mm in diameter). We then waited until tumors reached 1 cm in diameter (4–6 weeks later) and quantified solitary DCCs and metastatic lesions in lungs (Fig. 4a, Supplementary Table 4). We found that the time to tumor detection was slightly delayed when macrophages were depleted during asymptomatic pre-malignant stages (Fig. 4b, Supplementary Table 4). However, once palpable tumors had formed, the progression to overt tumors was not affected (Fig. 4c, Supplementary Table 4). Additionally, overt tumors showed no difference in macrophage content (Fig. 4d–f, Supplementary Table 4) and vascularization (Fig. 4g–i, Supplementary Table 4) at the end of the experiment between control-treated and anti-CSF1R-treated mice. This suggests that there is no impact on overt tumor growth in late tumors when macrophages were depleted during early stages of cancer progression. Additionally, flow analysis of lungs revealed that neither alveolar macrophage nor CD11b^+^/Gr1^+^ monocyte content was affected (Supplementary Fig. 3B–F, Supplementary Table 7) by the same treatment, arguing against a non-specific depletion of macrophages. However, CSF1R blockade during early stages significantly (*p* = 0.028) decreased solitary DCC burden in lungs (Fig. 4j, Supplementary Table 4). CSF1R blockade during early stages also caused a statistically significant (*p* = 0.039) decrease in the number of metachronous metastases per mouse (Fig. 4k–l, Supplementary Table 4), which were defined as the number of metastatic lesions bigger than three cells (Fig. 4k–l, Supplementary Table 4). Previous work^9^, which was confirmed here, showed that DCC clusters >5 cells tend to be positive for proliferation markers (P-Rb and P-Histone-H3), arguing that they are growing micrometastasis. This inhibitory effect of DCC burden and metastasis was detected even after macrophage depletion had been stopped in average for 1 month and animals had carried fast-growing tumors. We conclude that macrophages aid early dissemination of HER2^+^ early cancer cells, allowing for the early DCCs to reach target organs and form metastasis.

### Profile of myeloid cells in HER2^+^ early lesions

**Fig. 5:**
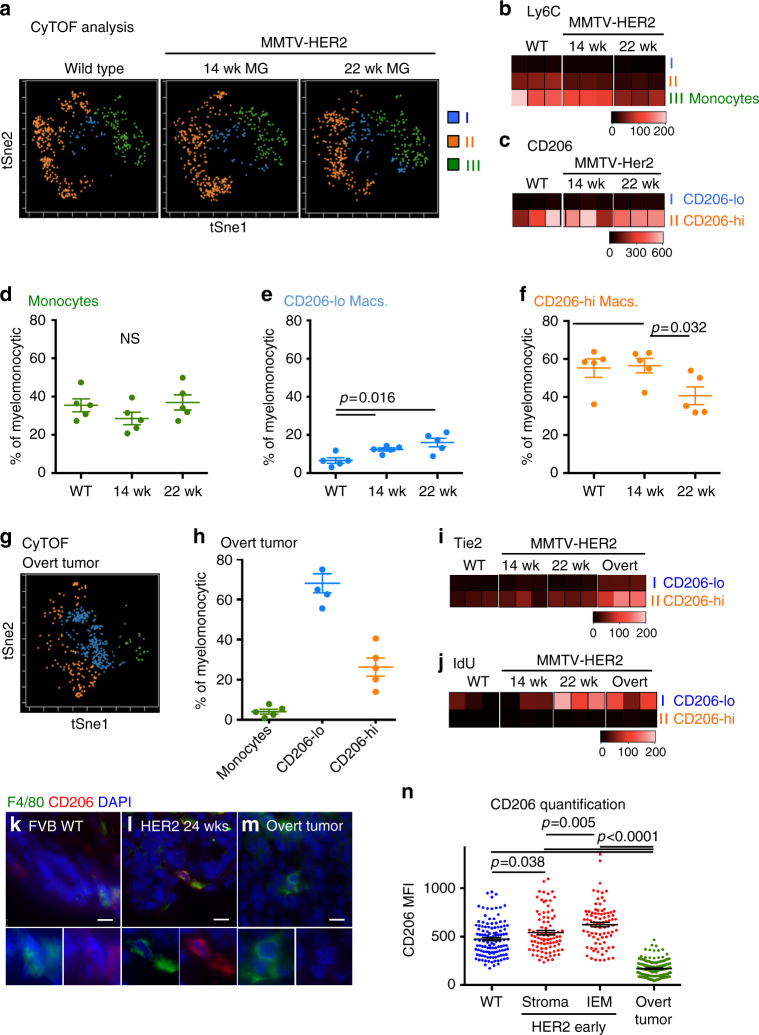
Phenotypic profiling of immune cells in early mammary cancer lesions. **a** Whole mammary glands from FVB wild-type mice or 14-week-old and 22-week-old pre-malignant MMTV-HER2 mice were analyzed by mass cytometry. viSNE plots were generated from myelomonocytic cells (gating strategy see Supplementary Fig. 4A, B). Results from one representative animal is shown; number of animals per group *N* = 5; individual experiments *N* = 2. The three sub-populations were identified as Ly6C^+^ monocytes and CD206^hi^ and CD206^lo^ macrophages based on their expression levels of Ly6C (**b**) and CD206 (**c**). These three populations were then analyzed for their frequency amongst all myelomonocytic cells. **d**–**f** Dot plots show mean±SEM of five animals per group. Heat plots for three individual animals per group with expression levels of selected markers Ly6C (**b**), CD206 (**c**), Tie2 (**i**), and IdU incorporation as a proliferation marker (**j**) were generated for CD206^lo^ and CD206^hi^ macrophages as identified in the viSNE plots. **g**, **h** viSNE plot and quantification of myelomonocytic population in overt MMTV-HER2 tumors (five mice per group). Mammary glands from FVB wild-type mice (**k**, 22wks), MMTV-HER2 mice at 24wks (**l**) and overt MMTV-HER2 tumors (**m**, 26–30wks) were stained against CD206 and F4/80 and CD206 signal intensity in F4/80^+^ macrophages was quantified; zoom factor in **k**, **l**, and **m**, 2x. Plot **n** depicts mean±SEM, each dot represents one macrophage; three animals combined. All bars: 10 μm. All statistical testing was done with 95% confidence interval by Mann–Whitney test

We next performed an unbiased profiling of macrophages and other immune cells present in WT and early lesions using time-of-flight mass cytometry (CyTOF) (Fig. 5a–c, Supplementary Table 5) and conventional fluorescence-activated cell sorting (FACS) (Supplementary Fig. 4A–G). Our data in the MMTV-Her2 model parallel the differences between WT mammary gland-associated and tumor-associated macrophages described previously in the MMTV-PyMT model^35^. Our CyTOF analysis^36^ showed that frequency and expression patterns of different myelomonocytic cells was similar between WT glands and early lesions, but differed from overt tumors (Fig. 5a, Supplementary Table 5, Supplementary Fig. 4A, B, and Supplementary Table 7). Monocytes were identified based on their high Ly6C levels (Fig. 5b, Supplementary Table 5) and their percentage relative to CD45^+^ cells increased in early lesions compared to WT mammary tissue, but this was not due to the increase in numbers (Fig. 5b, d, Supplementary Table 5, and Supplementary Fig 4F). The remaining LY6C^neg^ macrophages were distinguished based on CD206 expression (CD206^hi^ and CD206^lo^). CD206^lo^ macrophages slightly increased in early lesions compared to WT mammary tissue as detected by CyTOF and multicolor FACS (Fig. 5c, e, Supplementary Table 5); CD206^hi^ macrophages were more frequent in WT and early lesions and did not change between WT and early lesions, but decreased in frequency in overt tumors (Fig. 5c, f, Supplementary Table 5, Supplementary Fig. 4E, Supplementary Table 7). The changes in frequency for CD206^hi^ and CD206^lo^ were not due to an increase in total macrophage numbers (Supplementary Fig. 4E, Supplementary Table 7). Iododeoxy-uridine (IdU) incorporation analysis revealed that CD206^lo^ macrophages were more proliferative starting at week 14 throughout to over tumor stages, while CD206^hi^ macrophages remain non-proliferative (Fig. 5j, Supplementary Table 5). In early lesions CD206^hi^ macrophages expressed slightly higher levels of CD11b than CD206^lo^ macrophages (Supplementary Fig. 4C). Tie2 levels were significantly (*p* < 0.05) different between CD206^hi^ and CD206^lo^ macrophages in either CyTOF or conventional FACS measurements (Fig. 5i, Supplementary Table 5, Supplementary Fig. 4G, Supplementary Table 7). We observed no changes in other lymphoid or myeloid cells (Supplementary Fig. 4F, Supplementary Table 7). Overt tumors, as expected, showed changes in almost all populations (Supplementary Fig. 4F, Supplementary Table 7). We next performed in situ staining to define the phenotype and location of intra-epithelial macrophages in early lesions, which cannot be resolved by FACS (Fig. 5k–m, Supplementary Table 5). We found that stromal and intra-epithelial macrophages in both WT glands and early lesions displayed higher CD206 mean fluorescence intensity than that detected in macrophages in overt tumors (Fig. 5n, Supplementary Table 5). Our data suggest that circulation-derived monocytes do not contribute to the CD206 macrophage pool at early lesions. However, the IF analysis show that CD206^hi^ macrophages are the macrophage subset found within the stroma and intra-lesion compartments in early lesions, but that intra-lesion macrophages express the highest CD206 levels, suggesting a local translocation (Fig. 5k–n, Supplementary Table 5).

### Pre-malignant cells attract macrophages via CCL2

**Fig. 6:**
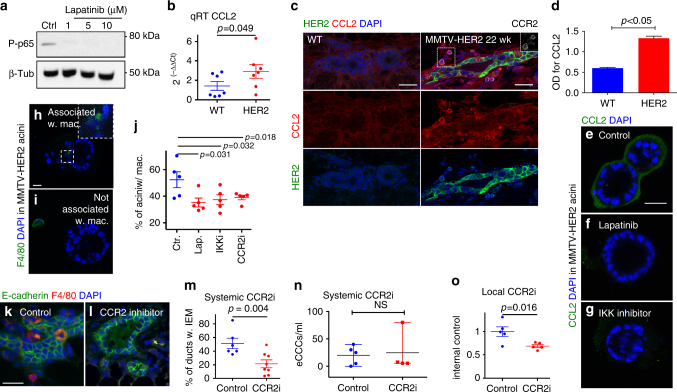
HER2 activates NF-κB and upregulates CCL2. **a** Western blot for NF-κB subunit phospho-p65 in mammosphere (MS) lysates from 20-week-old MMTV-HER2 mice. One representative blot of three independent experiments is shown. **b** CCL2 expression in MS from FVB wild-type and pre-malignant MMTV-HER2 mammary glands (MGs). Technical replicates, three experiments (3 mice/group); statistical analysis: with 95% confidence interval by Mann–Whitney test. **c** MGs from FVB wild-type and 22-week-old MMTV-HER2 mice stained against CCL2, HER2, and CCR2. Bars: 25 μm. Inset, CCR2 signal, zoom factor 1x; full image in Supplementary Fig. 5i. CCL2 intensity was quantified using ROI tool in Metamorph (Supplementary Fig. 5h). **d** ELISA of CCL2 in 24 h conditioned media of WT or HER2^+^ MS isolated from two animals. *P* value with 95% confidence interval by Mann–Whitney test—SEM shown. **e**–**g** CCL2 staining in acini cultures from MMTV-HER2 MGs (20–24 weeks (wks)) grown for 5 days and then DMSO-treated (vehicle control; **e**), 1 μM lapatinib (**f**) or 1 μM IKKi compound A (**g**) for 24 h. Bar: 25 μm. **h**–**j** MG acini treated with DMSO (vehicle), lapatinib (1 μM), IKKi compound A (1 μM), or CCR2 inhibitor RS504393 (1 μM). Primary MG macrophages were then added and the percentage of acini associated with or without macrophages (**h**) was quantified (**i**; bar: 25 μm; zoom factor 2.6x); **j** mean±SEM; each dot one technical replicate; two independent experiments; statistical analysis: Mann–Whitney test—SEM shown). **k**–**m** Twenty-week-old MMTV-HER2 mice were treated with CCR2 inhibitior RS504393 (2 mg/kg i.p. daily) for 2 weeks and stained against E-cadherin and F4/80 (**k**, **l**; bars: 25 μm). **m** Intra-epithelial macrophage (IEM) containing ducts were quantified (with 95% confidence interval by Mann–Whitney test; each dot: mean±SEM of one animal). **n** Quantification of circulating cancer cells (CCC) in the mice in the experiment in **k**–**m** (statistical analysis: Mann–Whitney test—SEM shown). CCC quantification was performed as in Fig. 3h. **o** Twenty-week-old MMTV-HER2 mice were treated locally with 1 mg/kg CCR2 inhibitor injected into the MG fat pad every day for 5 days and vehicle control into the contra-lateral gland. Glands were stained against F4/80 and IEM containing ducts were quantified (**o:** 5 mice per group; statistical analysis: Mann–Whitney test—SEM shown). Values in **o** normalized to the IEM content of each contra-lateral control-treated gland

We next focused on what signals might HER2 upregulation induce to attract local macrophages from the stroma into the early lesions. In invasive breast cancer models, HER2 signaling activates NF-κB, which transcriptionally induces CCL2, a potent macrophage chemotactic factor^37^. We found that the p65 subunit of NF-κB subunit was phosphorylated in mammospheres derived from HER2^+^ early cancer cells, and lapatinib, a HER2 and EGFR inhibitor, inhibited its phosphorylation (Fig. 6a, Supplementary Table 6 and Supplementary Fig. 7). We then isolated RNA from mammospheres derived from either FVB WT or MMTV-HER2 early lesions as described^9^ and performed quantitative real-time PCR (Q-RT-PCR) analysis for cytokine mRNAs. We found that already at these early stages of progression, HER2^+^ cancer cells upregulated expression of *Ccl2* but not *Csf*2, *Csf1*, *Il1β*, and *Il6* (Fig. 6b, Supplementary Table 6 Supplementary Fig. 5A, B, Supplementary Table 7). *Csf2* mRNA was detectable in WT and early lesion-derived spheres but the expression was not different between these groups and could not be confirmed by IF analysis (Supplementary Fig. 5C, E), while overt tumors showed detectable CSF2 arguing that early lesions do not express CSF2 (Supplementary Fig. 5F, Supplementary Table 7). Upregulation of CCL2 around HER2^+^ early cancer cells was also observed at the protein levels as confirmed by quantifying soluble CCL2 in the conditioned media of the early cancer cells by enzyme-linked immunosorbent assay (ELISA) and by IF analysis (Fig. 6c, d, Supplementary Table 6 and Supplementary Fig. 5G–H, Supplementary Table 7). Quantification of the increase in CCL2 IF signal (Supplementary Fig. 5H, Supplementary Table 7) in the HER2^+^ cells vs. WT epithelium in Fig. 6c (see also, Supplementary Table 6) revealed a difference that was confirmed by ELISA and quantitative PCR (qPCR) from isolated mammospheres from the same primary tissues. CCR2^+^/CCL2^+^ cells could be found close to CCL2^+^ early cancer cells that showed lower signal for both CCR2 and CCL2; these signals were undetectable in WT tissues (Fig. 6c insert shows CCR2, Supplementary Table 6, Supplementary Fig. 5I, Supplementary Table 7). Q-RT-PCR of RNA isolated from FACS-sorted monocytes, macrophages, neutrophils, and HER2^+^ cancer cells during early stages of progression confirmed the CCL2 production by HER2^+^ early lesions (Supplementary Fig. 5J, Supplementary Table 7), as detected by q-RT-PCR, IF analysis, and ELISA (Fig. 6b–d, Supplementary Table 6). HER2^+^ early lesions produce lower levels of CCL2 mRNA than monocytes and macrophages, suggesting multiple sources of CCL2. Organoids produced by early MMTV-HER2 cancer cells displayed a reduction in secreted and peri-organoid CCL2 production upon inhibition of HER2 or NF-κB signaling with specific inhibitors^38^ as detected by IF analysis and ELISA (Fig. 6e–g, Supplementary Table 6 and Supplementary Fig. 5G, K, Supplementary Table 7). To confirm that CCL2 signaling was necessary and sufficient for HER2-dependent macrophage attraction, HER2^+^ early cancer cells were grown as three-dimensional (3D) acinar structures in vitro for 5 days. Cultures were then treated with lapatinib, an IKKβ inhibitor,^38^ or an inhibitor of the CCL2 receptor, CCR2 (RS504393), and macrophages isolated from mammary glands of MMTV-HER2 mice were added to the cultures. After 24 h of co-culture, macrophages were associated with ~50% of all organoid structures in control samples (Fig. 6h, Supplementary Table 6). In contrast, co-cultures treated with the inhibitors all showed significant (*p* < 0.04) reduction in macrophage association (Fig. 6i, j, Supplementary Table 6). When we tested whether CCL2 was responsible for Wnt-1 production by macrophages, we found that Raw264.7 cells treated with recombinant CCL2 at doses known to stimulate chemotaxis^39^, produced more Wnt-1 than control-treated cells (Supplementary Fig. 5L). We next treated 20-week-old MMTV-HER2 mice carrying only early lesions (no palpable or overt tumors) for 2 weeks with a CCR2 inhibitor (Fig. 6k, l, Supplementary Table 6). We found that the number of intra-epithelial macrophages was significantly (*p* = 0.004) reduced (~40%) when mice were treated systemically (Fig. 6m, Supplementary Table 6). However, systemic CCR2 inhibition did not reduce the number of early circulating cancer cells (eCCCs) (Fig. 6n, Supplementary Table 6) compared to the treatment that blocked the CSF1R, which reduced intra-epithelial macrophages by ~77% (Fig. 3h, Supplementary Table 6). It is possible that the CCR2 inhibitor is less potent in blocking macrophage translocation than the anti-CSF1R antibody as evidenced by the stronger inhibition in macrophage translocation into the mammary tissues. When the CCR2 inhibitor was administered locally into the fat pad to avoid more widespread systemic effects (Fig. 6n, Supplementary Table 6), intra-epithelial macrophage content was reduced compared to contra-lateral control-treated glands (Fig. 6o, Supplementary Table 6). We conclude that HER2 signaling in cancer cells from early lesions activates NF-κB to induce CCL2, but that other myeloid cells also produce significant CCL2 mRNA. We further conclude that CCL2 may be a signal that stimulates macrophages to produce Wnt-1 as shown in Fig. 2k–l.

### Intra-epithelial macrophage in patient DCIS lesions

**Fig. 7:**
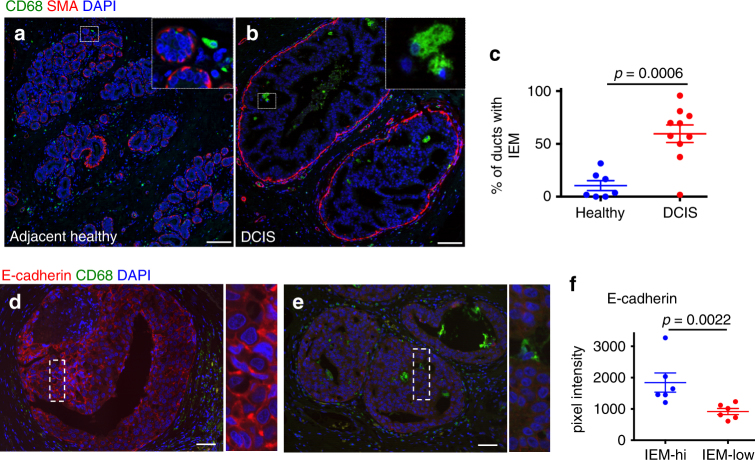
Intra-epithelial macrophage numbers in human DCIS lesions negatively correlate with E-cadherin levels. Human adjacent healthy (**a**) and DCIS tissue (**b**) was stained against CD68 (macrophages) and smooth muscle actin (SMA). Bar: 75 μm; zoom factor 4.75x. **c** Plot shows mean±SEM of the percentage of ducts containing intra-epithelial macrophages (IEMs) from 7 healthy and 10 DCIS patients; each dot represents one patient; statistical analysis: Mann–Whitney test. Sections from human DCIS tissue were stained against CD68 (macrophages) and E-cadherin (**d**, **e**, bar: 75 μm; zoom factor 4.1x). E-cadherin pixel intensity was quantified in regions of individual cell junctions and medians for individual patients were quantified. Plot **f** depicts mean E-cadherin intensity throughout DCIS lesions of individual patients with low or high intra-epithelial macrophage (IEM) numbers (total patient number *N* = 12; statistical analysis: Mann–Whitney test—SEM shown)

Published data showed that more than 10% of patients with DCIS had detectable DCCs in their bone marrow (BM), but no histologic markers, which include invasive fronts and receptor status were indicative of the presence of DCCs^3^. Macrophages are detected in the stroma of normal breast tissue^40^. To test whether macrophages could also infiltrate early lesions in humans, we undertook a pilot analysis of a small number of patient samples and compared macrophages in healthy human breast tissue vs. tissue from DCIS lesions as a model of early-stage breast cancer lesions. Macrophages were identified as CD68^+^/CD45^+^ and CK8/18^−^ cells (Supplementary Fig. 6A, B). As reported^40^, in breast tissue from healthy donors (*n* = 7), CD68^+^/CD45^+^/CK8/18^−^ macrophages were localized in the stroma in the vicinity of mammary ducts but remained outside the ducts, which were delimited by an intact myoepithelial layer of cells (Fig. 7a). In contrast, even in DCIS lesions that displayed an apparently intact myoepithelial layer, there was a statistically significant (*p* = 0.0006) increase in the frequency of macrophages found inside the aberrant ductal epithelial structure in between cancer cells (*n* = 10) (Fig. 7b, c). The intra-epithelial CD68^+^/CD45^+^/CK8/18^−^/E-cadherin^−^ macrophages were commonly associated with cancer cells (CD68^−^/CD45^−^) that showed reduced E-cadherin levels in human DCIS samples (*N* = 12) as measured by quantitative image analysis (Fig. 7d–f). Additionally, within the same patient, individual lesions with high macrophage numbers had lower E-cadherin levels (Supplementary Fig. 6C–E). Additional breakdown in grade subgroups showed that healthy tissues and low-grade DCIS showed no difference in the frequency of intra-lesion macrophages (Supplementary Fig. 6F). However, high-grade lesions showed a significant (*p* < 0.04) increase in intra-lesion macrophages when compared to healthy and low-grade tissue (Supplementary Fig. 6F). Quantification of intra-lesion macrophages in HER2^−^ or HER2^+^ DCIS lesions revealed no differences (Supplementary Fig. 6G). This work is limited by the low number of patients. However, the pilot data suggest that other oncogenic signals associated with a high-grade histological subtype in humans could also result in macrophage attraction into the lesions. These may be signals as those propagated by the PyMT oncogene as macrophages in TMEM structures were detected in early MMTV-PyMT lesions (Supplementary Fig 1C–E and 2A).

## Discussion

Our published work has shown that oncogene-driven early dissemination is due to the co-option by oncogenic pathways of mechanisms of motility and invasion activated during mammary branching morphogenesis^8, 9^. This included the activation of cell intrinsic pathways that through an EMT-like program propelled early dissemination and metastasis^8, 9^. Importantly, early dissemination was documented in humans in breast and pancreatic cancer^6, 41^. Our data suggest that macrophages enter the epithelium of early lesions in mice where they create early dissemination microenvironments that in both the PyMT and HER2 models had all the components of TMEM^21, 33^. Based on our data we hypothesize that HER2^+^ early cancer cells attract macrophages locally and in response to the CCL2 signal these macrophages also produce CCL2, as macrophages from WT mammary glands expressed lower levels of CCL2. We further hypothesize that these intra-epithelial macrophages (IEM) macrophages produce Wnt-1 in response to CCL2 to dismantle epithelial E-cadherin junctions. That the CCR2 inhibitor did not result in a decrease in eCCCs may be due to dosing limitations or to a stronger dependence of eCCC precursors on CSF1 than CCL2. Nevertheless, our in vitro 2D and 3D organoid culture data allow us to propose that, while early cancer cells produce several Wnt ligands^8, 9^, Wnt-1 may be exclusively produced by macrophages. However, we acknowledge that a limitation of our study is the absence of *in vivo* validation of the Wnt-1 role in the context of early dissemination. The dissemination-promoting function of macrophages was proven when we found that depletion of macrophages in early HER2^+^ lesions using anti-CSF1R antibodies reversed the loss of E-cadherin in HER2^+^ lesions as well as intravasation and dissemination to lungs. Interestingly, Wnt signaling is linked to branching morphogenesis^42, 43^ and a subset of tumor-associated macrophages that drive invasive cancer dissemination through TMEM formation also secrete Wnt ligands^31^, while CCL2 production by colorectal cancer cells can also foster vascularization and intravasation as shown in more advanced tumors^44^. The detection of TMEM in the PyMT and HER2 early lesions not only support that these mechanisms are not HER2 exclusive but also that these structures that orchestrate intravasation and have a clinical prognostic value^33^ may be operational at earlier time points than expected from the invasive cancer paradigm.

We found that the myelomonocytic landscape of HER2^+^ early lesions at the time of early dissemination resembled that of healthy mammary glands and paralleled the findings in the PyMT model^35^. Early lesions contained both F4/80^+^/CD11b^+^/CD206^hi^/Tie2^hi^ and F4/80^+^/CD11b^+^/CD206^lo^/Tie2^lo^ macrophages. In contrast, overt tumors predominantly contained CD11b^int^/CD206^lo^/Tie2^hi^ macrophages as described^35^. In situ imaging supported that F4/80/CD206^hi^ macrophages may be more frequently intra-epithelial but the total number and proportion in the mammary tissue do not differ from the CD206^lo^ population. That CD206^hi^ populations of macrophages were Tie2^hi^ suggest that they might function as a subset of macrophages in invasive breast cancer lesions that are gatekeepers of intravasation doorways^21^. Our results raise the possibility that when HER2 or other oncogenic signals are activated in mammary epithelial cells, macrophages that translocate into the early lesions from the stroma have the inherent potential to aberrantly fuel epithelial cell motility as observed during mammary gland development^19, 20, 45^. However, further scrutiny and lineage tracing experiments would be required to fully understand the origin of macrophages driving early dissemination.

**Fig. 8:**
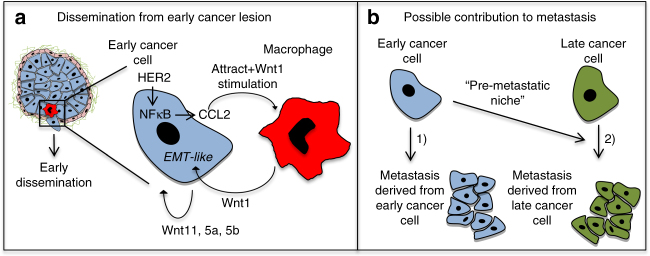
Models summarizing findings and potential scenarios for early DCC role in metastasis development. **a** Scheme of macrophage-regulated early dissemination from pre-invasive lesions where CCR2^+^/CD206^hi^/F4/80^hi^/Tie2^hi^ macrophages are attracted into early lesions by HER2-mediated and NF-κB-mediated upregulation of CCL2. Our models support that intra-epithelial macrophages in turn secrete Wnt-1 in response to CCL2 production by cancer cells and also other immune cells and thereby further cement an EMT-like response that is also stimulated by autocrine/paracrine production of other Wnt ligands^8, 9^ to drive early dissemination. **b** Early DCCs contribute to metastasis formation, either as a slow cycling seeds of metastasis (scenario 1) or by interacting with the microenvironment and/or later arriving DCCs to create a “pre-metastatic niche” that is eDCC-orchestrated and more permissive for the growth of late or early and late cancer cells (scenario 2)

We found that when macrophages were depleted during early stages, but allowed to rebound during invasive stages, lung metastatic burden was reduced. This result indicates that early DCCs contribute to lung metastasis formation, either directly or indirectly. Surprisingly, large tumors that persisted in mice for ~1.5 months were not able to compensate for the reduced macrophages and dissemination during early stages. This may be due to the lower dissemination capacity we observed in HER2^+^ overt tumors vs. early lesions^8^. Further, that the number of solitary DCCs, likely a mixture of DCCs accumulated since the early stages, was reduced by CSF1R blockade suggests that the reduced influx of DCCs to lungs during early stages was not replaced by DCCs arriving during the time of tumor detection to euthanasia when tumors were large. This is in agreement with our recent phylogenetic data that a fraction of metastasis detected late in MMTV-HER2 animals were indeed derived from early DCCs and not from the overt primary tumor^8^ (Fig. 8a, scenario 1). Additionally, early DCCs may cooperate with later arriving DCCs to form metastasis in patients, which after DCIS treatment go on to develop invasive lesions or in patients who had DCIS but only were diagnosed later for invasive cancer (Fig. 8b, scenario 2). The low number of patients in our pilot analysis and the fact that there might be mouse to human differences in macrophage function limits the conclusions of our study on the human relevance of our findings. Nevertheless, it encourages further testing of human samples to determine whether detection of macrophages and E-cadherin levels in DCIS lesions may help predict which patients have early dissemination.

Early dissemination may also impact the concept of the “pre-metastatic” niche^46, 47^ where this microenvironment could be orchestrated by early DCCs in addition to BM-derived cells. Qian et al.^26^ showed that in MMTV-PyMT-invasive cancer models, VEGFR^+^/CCR2^+^/CXCR4^−^/Tie2^−^ macrophages fuel metastasis in the lung^26^. Our data show that macrophages rebound in tumors during overt growth stages. Still, even after this rebound, metastasis were reduced if the anti-CSF1R-mediated depletion of macrophages was done during early stages. We propose that the VEGFR^+^/CCR2^+^/CXCR4^−^/Tie2^−^ macrophage-mediated support of metastasis may not be able to compensate for the reduction in DCCs caused by macrophages depletion during early dissemination in primary sites. However, we cannot rule out the possibility that the anti-CSF1R monoclonal antibodies affected the population described by Qian et al.^26^ in lungs. Thus, it is possible that during early stages, depletion of VEGFR^+^/CCR2^+^/CXCR4^−^/Tie2^−^ macrophages also impacted early DCC lodging in lungs. This is an interesting possibility we will explore in future studies.

Overall, our studies suggest that oncogenes might turn on developmental programs of anoikis resistance^28^, migration and invasion^8, 9^, and macrophage translocation into the early lesions much earlier than anticipated. These programs then initiate early dissemination before propelling rapid tumor growth. Overall, in this study, we provide critical new insights into the understanding of the natural history of metastatic disease in mice and we demonstrate that early in cancer evolution macrophages and early DCCs appear to play a seminal role in metastatic breast cancer that can manifest later in life of these mice.

## Methods

### Cells and cell culture

Raw264.7 cells were received from ATCC. Transfection with mCherry was done using mCherry lentiviral vectors and maintained in DMEM (Lonza) with 10% fetal bovine serum (FBS) and 1% Pen/Strep. Comma-1D cells were a generous gift from Daniel Medina and were maintained in Dulbecco's modified Eagle's medium: Nutrient Mixture F12 (DMEM-F12) medium containing 2% FBS and 1% Pen/Strep. For DKK1 stimulation, conditioned media were prepared from DKK1-expressing 293T cells which were a gift from S. Aaronson. For preparation of conditioned medium (CM), protein concentration was determined using a Bradford assay; cells were cultured with serum-free medium (DMEM + 1% Pen/Strep) for 24 h and then concentrated using Vivaspin 20 Centrifugal Concentrating tubes (Sartorius, VS2021) at 3000×*g* up to 3 h until a desired concentration (10x) was reached. DKK1 protein (0.5 μg/ml) was used for stimulation. For co-culture experiments, Comma-1D cells were seeded on coverslips, and after 12 h, Raw264.7-mCherry cells were added. Co-cultures were fixed in 2% formalin after 12 h and then stained. All cell lines were routinely tested for mycoplasma. Raw264.7-mCherry cells were treated with recombinant CCL2 (20 ng/ml, catalog number: 250-10, Peprotech), and 24 h later, the levels of Wnt-1 were detected by qPCR. Comma-1D cells were cultured with the CM from Raw264.7-mCherry cells (24 h, diluted 1/40), and after 24 h, the percentage of cells forming E-cadherin junctions were detected by IF analysis. Raw264.7-mCherry CM was incubated overnight with anti-WNT1 ab (ab15251, Abcam) or IgG control, then depleted from CM and finally added to Comma-1D cells.

### Mouse experiments

All animal procedures were approved by the Institutional Animal Care and Use Committee (IACUC) of Icahn School of Medicine at Mount Sinai, protocols 08-0366 and 2014-0190. Mice used were female FVB/N-Tg(MMTVneu)202Mul/J or FVB WT mice and were purchased from Jackson Laboratory and bred in-house. Animals were killed using CO_2_ at age 14 weeks, 20–22 weeks or when invasive tumors had reached a diameter of 1 cm. For macrophage depletion, we administered 3 mg of the CSF1R antibody clone ASF98 on day 1 and 1 mg on day 7 and weekly thereafter by injection into the tail vein of 18-week-old pre-malignant MMTV-Her2 mice. PBS was used as a vehicle control. Treatment lasted 14 days or until tumors were first palpable (3 mm diameter). ASF98 antibody was a generous gift from Dr. Miriam Merad. For CCR2 blockade, mice were either treated with 2 mg/kg of RS504393 (Tocris Bioscience) or vehicle control (dimethyl sulfoxide (DMSO)) daily by intraperitoneal injections for 14 days or by injection of 1 mg/kg RS504393 into the fat pad for 5 days.

### Mammary gland whole mounts

Mammary glands were excised, spread on glass slides and fixed in overnight in 4% formalin at 4 °C. Glands were then dried for 5 min and fixed in Carnoy’s fixative (60% ethanol, 30% chloroform, 10% glacial acetic acid) for 2–4 h at room temperature. Glands were washed in 70% ethanol for 15 min and in 50% ethanol twice for 15 min each and rinsed with distilled water for 5 min. Staining with carmine alum solution occurred overnight (0.2% carmine, 0.5% aluminum potassium sulfate in water boiled for 20 min and then filtered). Glands were washed in 70% ethanol for 15 min, in 96% ethanol and in 100% ethanol for 15 min and in xylene for 15 min and stored in methyl salicyate.

### Microscopy

Formalin-fixed and paraffin-embedded samples were prepared and stained as described^48^. For IHC, VectaStain Elite ABC Rabbit IgG and Mouse IgG Kits (PK-6102) from Vector Laboratories were used for secondary antibodies. Secondary antibodies were left for 1 h at room temperature. DAB and Vector Blue Substrate Kit (Vector Laboratories) were used for enzymatic substrate. Mounting was done using VECTASHIELD Mounting Media (Vector Laboratories). For CD206 stainings, cryosections were used. Tissue was fixed in 4% formalin overnight, incubated in 30% sucrose/PBS overnight and sectioned into 6 μm sections. Staining of cryosection was done as described^49^. Antibodies used were: CD68 (Sigma, #MS397-PO, 1:100), CD45 (Cell Signaling, #13917P, 1:100), F4/80 (Abcam, #ab6640, CIA:3-1, 1:100), Iba1 (Wako, #019-19741, 1:200), CK8/18 (Progen, #GP11, 1:200), smooth muscle actin (Sigma, #A2547, clone IA4, 1:200), CD206 (BioLegend, #141073, clone 068C2, 1:100), CCL2 (Novus, #NBP2-22115, clone 2D8, 1:100), CCR2-647 (BioLegend, #150603, clone SA203G11, 1:50), E-cadherin (Becton Dickinson, #610181, clone 36/E, 1:100), β-catenin (Cell Signaling, #8480S, clone D10A8, 1:100), Her2 (Abcam, #ab2428, 1:100), and endomucin (Santa Cruz, #sc-65495, clone 7C7, 1:100). For co-staining of F4/80 and CD206 (both raised in rat), an A488-conjugated F4/80 antibody (BioLegend, #123119, clone BM8, 1:50) was used in combination with CD206 (BioLegend, #141726, clone C068C2, 1:100) in a sequential stain. Microscopic analysis was carried out with a Leica widefield microscope or with a Leica confocal microscope for 3D cultures. For quantification of IF signal intensity with the Metamorph software, regions of interest were defined in original tiff files that had been taken under the same exposure time and settings and the mean signal intensity was measured. Because the use of a directly conjugated F4/80 antibody resulted in lower signal intensity, we used Iba1 as a macrophage marker^21^ to identify macrophages for CD206 signal intensity measurement instead. For detection of micrometastasis and macrometastasis we followed published protocols^9^. Briefly, the dormant non-proliferative lesions are mostly single cells or clusters of up to 10 cells as defined by the absence of proliferation markers such as Ki-67, P-Rb and P-ser10-histone H3^9, 48, 50, 51^. In some studies, we found an upper limit of 20 cell clusters completely devoid of proliferation makers. For each study, the analysis of proliferative vs. non-proliferative lesions is calibrated for a specific operator that is developing the study. In this paper, we found that 5-cell clusters were a good indication of a lower limit for what we call a proliferative micrometastasis. This number was within range of our previous studies in the MMTV-Her2 model^9^.

### Flow cytometry

MMTV-HER2 mice were killed using CO_2_ at age 18–22 weeks (early pre-malignant cancer lesions) or when overt tumors had formed. Whole mammary glands or tumors were digested in collagenase/bovine serum albumin (BSA) at 37 °C for 30–45 min. Red blood cell lysis buffer (Sigma) was used to remove blood cells. Cell suspensions were blocked with Fc-blocking reagent (eBioscience) and samples were surface stained in FACS buffer (PBS supplemented with 1% BSA and 2 mM ethylenediaminetetraacetic acid) for 20–30 min on ice using the following antibodies: CD45-PerCPCy5.5 (BioLegend, #103131, clone 30-F11, 1:200), CD11b-PeCy7 (eBioscience, #101215, clone M1/70, 1:200), CD11c-PE (eBioscience, #117307, clone N418, 1:100), Gr1-AF700 (eBioscience, #56-5931-80, clone RB68C5, 1:200), Tie2-biotin (eBioscience, #13-5987-82, clone TEK4, 1:100), F4/80-biotin (BioLegend, #123105, clone BM8, 1:100), CD206-APC (BioLegend, #141707, clone C068C2, 1:100), VCAM-FITC (eBioscience, #11-1061-82, clone 429, 1:50). DAPI (4',6-diamidino-2-phenylindole) was used to label dead cells. Multiparameter analysis was performed on a Fortessa (BD) and analyzed with the FlowJo software (Tree Star). DAPI^+^ cells and doublets were excluded from all analysis. To sort mammary tissue macrophages, whole mammary glands from 14-week to 18–22-week (early pre-malignant cancer lesions) mammary glands or from invasive tumors were digested in collagenase/BSA at 37 °C for 30–45 min. Mononuclear cells were enriched in a Percoll gradient and then macrophages were sorted as viable CD45^+^/Gr1^−^/CD11b^+^/F4/80^+^ cells.

To study the myeloid and lymphoid mammary gland infiltrate, FVB/N WT, MMTV-HER2 18–22 weeks and MMTV-HER2 females with overt tumors were digested as above and incubated with a panel for myeloid markers: Tie2-PE (eBioscience, #12-5987-82, clone TEK4, 1:200), F4/80 APC (BioLegend, #123115, clone BM8, 1:200), CD11c PECy7 (eBioscience, #25-0114-82, clone N418, 1:200), MHCII AlexaFluor700 (eBioscience, #56-5321-82, clone M5/114.15.2, 1:100), CD11b PerCPCy5.5 (eBioscience, #45-0112-82, clone M1/70, 1:200); CD206 FITC (BioLegend, #141704, clone C068C2, 1:200); CD106-biotin (eBioscience, #13-1061-82, clone 429, 1:200), streptavidin APC eFluor780 (eBioscience, #47-4317-82, 1:500); Ly6G eFluor450 (Biolegend, #127612, clone 1A8, 1:200) and lymphoid markers (CD8 APC, eBioscience #17-0081-82, clone 53-67, 1:200; CD4 PECy7, eBioscience #25-0041-82, clone GK1.5, 1:200; CD3 PerCPCy5.5, eBioscience #45-0031-82, clone 145-2C11, 1:200; B220 FITC RA3-62B, eBioscience #11-0452-82, 1:200; CD19 eFluor450, eBioscience #48-0193-82, clone eBio1A3, 1:200). Counting beads (40,000 beads/ml) were added per tube (500 μl per tube) and 2000 beads were acquired of every sample (AccuCheck Counting Beads Life Technologies, PCB100). Absolute numbers of CD45+ leukocytes were quantified as follows:

$${ \frac{{\rm No. of\,cells}}{{\rm No. of\,cells\,acquired}}} \times {\frac{{\rm No. of\,beads}}{{1\,\rm ml}}}{\times}{\frac{{0.5\,\rm ml}}{{\rm tube}}} = \ {\rm No. of\,cells/tube}$$

Percentages obtained in flow cytometry among CD45^+^ cells (calculated as mentioned before) were used to obtain absolute cell counts of every population.

### Sorting of myeloid and CD45^neg^ Her2^+^ populations

Mammary glands from MMTV-Her2 females between 18 and 22 weeks old were digested as mentioned above. Mammary tissue was incubated with a cocktail of antibodies including CD11b-eFluor450 (eBiosciences, #48-0112-82, clone M1/70, 1:200), CD45-BV510 (BioLegend, #103137, clone 30-F11, 1:200), Ly6C FITC (BD Biosciences, #553104, clone AL-21, 1:200), Ly6G-PE (BioLegend, #127607, clone 1A8, 1:200), CD11c APC eFluor780 (eBiosciences, #47-0114-82, clone N418, 1:200), F4/80 PECy7 (BioLegend, #123113, clone BM8, 1:200), and Her2 (Abcam, #ab2428, 1:200) antibody. Secondary anti-rabbit AlexaFluor647 (#A-21245, Invitrogen) was used for Her2^+^ cell detection at a 1:200 concentration. Cells were incubated for 30 min at 4 °C in dark, and subsequently washed and incubated for 30 more minutes with secondary antibody. PI (V35117, Life Technologies) was used as viability dye in 1:200 concentration. Monocytes were defined as CD45^+^CD11b^+^Ly6C^+^, neutrophils as CD45^+^CD11b^+^Ly6G^+^, and macrophages as CD45^+^CD11c^−^F480^+^CD11b^+^. CD45^−^ fraction was subsequently analyzed for Her2 expression. CD45-Her2^+^ cells were sorted. Sorted cells were directly lysed in Trizol LS (Ambion, 10296028) for RNA extraction.

### CyTOF analysis

All mass cytometry reagents were purchased from Fluidigm Inc. (former DVS), unless otherwise noted. Mice were injected intraperitoneally with 1 mg IdU per mouse 16 h prior to the experiment. Lymph nodes were removed and mammary glands were digested using the Miltenyi Fatty Tissue Digestion Kit. Cells were then washed with PBS containing 1% BSA and blocked with Fc-blocking reagent (eBioscience) to minimize non-specific antibody binding. Cells were stained with a panel of metal-labeled antibodies against 20 cell surface markers (Fig. 5, Supplement Fig. 1A) for 30 min on ice and then washed. All antibodies were either purchased pre-conjugated to metal tags or conjugated in-house using MaxPar X8 Conjugation Kits according to the manufacturer’s instructions. After antibody staining, cells were incubated with cisplatin for 5 min at room temperature as a viability dye for dead cell exclusion. Cells were then fixed and permeabilized with a commercial fix/perm buffer (BD Biosciences) and stored in PBS containing 1.6% formaldehyde and a 1:4000 dilution of Ir nucleic acid intercalator to label all nucleated cells. Immediately prior to acquisition, cells were washed in PBS, and diH_2_O, and resuspended in diH_2_O containing a 1/10 dilution of EQ 4 Element Calibration beads. After routine instrument tuning and optimization, the samples were acquired on a CyTOF2 Mass Cytometer in sequential 10 min acquisitions at an acquisition rate of <500 events/s. The resulting FCS files were concatenated and normalized using a bead-based normalization algorithm in the CyTOF acquisition software and analyzed with Cytobank. FCS files were manually pre-gated on Ir193 DNA^+^ CD45^+^ events, excluding dead cells, doublets, and DNA-negative debris. Myeloid-derived cells were manually gated based on CD11c and CD11b expression and the gated myeloid populations were then analyzed using viSNE^36^ based on all myeloid phenotypic markers. Putative cell populations on the resulting viSNE map were manually gated based on the expression of canonical markers, while allowing for visualization of additional heterogeneity within and outside the labeled population bubbles.

### Mammospheres and 3D mammary primary epithelial cell cultures

Acini cultures were performed as described^52, 53^. eCCs (5 × 10^4^) were seeded in 400 μl Assay Medium in 8-well chamber slides coated with 40 μl of Matrigel (Corning). Acini formed at an efficiency of around 30 acini/1 × 10^4^ mammary epithelial cells plated. For macrophage co-cultures, primary tissue macrophages were added at a ratio of 500 per 1 × 10^4^ eCCs seeded to 5-day-old acini cultures. For inhibitory treatments, 5-day-old acini cultures were treated for 24 h with 1 μM lapatinib (LC Laboratories), 2 μM IKK inhibitor^38^ (generous gift from Dr. Albert Baldwin), 1 μM CCR2 inhibitor RS504393 (Tocris Bioscience) or DMSO as vehicle control. Cultures were then fixed for IF with 4% PFA. Mammosphere cultures were prepared as described^53^. To prepare CM, 5-day-old mammosphere culture were plated in serum-free DMEM medium and CM was harvested after 24 h. ELISA detection for CCL2 was done following the protocol provided by the manufacturer (catalog number: MJE00, R&D systems).

### Immunoblotting and qPCR

Immunoblotting was performed as described previously^54, 55^. Antibodies used were P-NF-κB p65 (Cell Signaling, polyclonal) and β-tubulin (Abcam, polyclonal). For expression analysis of FVB WT mammary epithelial cells or MMTV-Her2 eCCs, epithelial cells were isolated and grown as mammospheres for 5 days as described^53^. For expression analysis of Raw264.7 macrophages, cells were grown as monolayers in 6-well culture dishes and treated with CM for 24 h. RNA isolation was performed using Trizol (Life Technologies) or the RNeasy Kit (Qiagen) for MTMs. RT-PCR and qPCR were performed as described^48^. All samples were normalized to glyceraldehyde 3-phosphate dehydrogenase (GAPDH) expression and 2^−Δ^ ^Δ^ ^Ct^ values were calculated as described^56^. Primers were purchased from IDT. Primer sequences were as follows: mouse—GAPDH forward primer, 5′-AACTTTGGCATTGTGGAAGGGCTC-3′; GAPDH reverse primer, 5′-TGGAAGAGTGGGAGTTGCTGTTGA-3. E-cadherin forward primer, 5′-CAAGGACAGCCTTCTTTTCG-3′; E-cadherin reverse primer, 5′-TGGACTTCAGCGTCACTTTG-3′. Wnt-1 forward primer, 5′-CAGTGGAAGGTGCAGTTGCAG-3′; Wnt-1 reverse primer, 5′-CAGTGGAAGGTGCAGTTGCAGC-3′. CSF1 forward primer, 5′-CAACAGCTTTGCTAAGTGCTCTA-3′; CSF1 reverse primer, 5′-CACTGCTAGGGGTGGCTTTA-3′. CCL2 forward primer, 5′- GGCTGGAGAGCTACAAGAGG-3′; CCL2 reverse primer, 5′- GGTCAGCACAGACCTCTCTC-3′.

#### Patient samples

Paraffin-embedded sections from patients with DCIS were obtained from the Cancer Biorepository at Icahn School of Medicine at Mount Sinai (New York, NY, USA). Samples were fully de-identified and obtained with informed consent with Icahn School of Medicine at Mount Sinai Institutional Review Board approval, which indicated that this work does not meet the definition of human subject research according to the 45 CFR 46 and the Office of Human Subject Research.

### CCC and DCC detection

For CCC analysis, blood was drawn by cardiac puncture following IACUC protocols. CCCs were purified using negative Lineage Cell Depletion Kit (Miltenyi), fixed with 3% PFA for 20 min on ice and cytospin preparations were carried out by centrifugation of blood cells at 500 r.p.m. for 3 min using poly-l-lysine-coated slides (Sigma). CCCs were stained by IF against CK8/18 and HER2. CK8/18^+^ and HER2^+^ cells were counted and plotted per ml of blood. For DCC analysis in lungs, lung sections were stained against HER2. Complete lung sections were screened for HER2^+^ DCCs at x1000-fold magnification. From each animal, the average number of DCC per section from three individual lung sections was quantified.

### Statistical analysis

Unless noted otherwise, all the experiments presented in the manuscript were repeated at least three times with replicates of at least 3. Statistical analysis was done using the GraphPad Prism software. For all cell culture experiments (experiments with technical replicates), one-tailed Student *t* tests were performed. For mouse experiments, Mann–Whitney tests were used. Differences were considered significant if *p* values were ≤0.05.

### Data availability

The authors declare that all the data supporting the findings of this study are available within the article and its supplementary information files and from the corresponding author upon reasonable request.

## Electronic supplementary material

Supplementary Information
