# Supplementary material for: Macrophages orchestrate breast cancer early dissemination and metastasis
Source: Nat Commun. 2018 Jan 2;9:21. doi: 10.1038/s41467-017-02481-5 (PMC5750231; doi:10.1038/s41467-017-02481-5)
Supplement: Supplementary file 1 — Supplementary Information [file 41467_2017_2481_MOESM1_ESM.pdf]

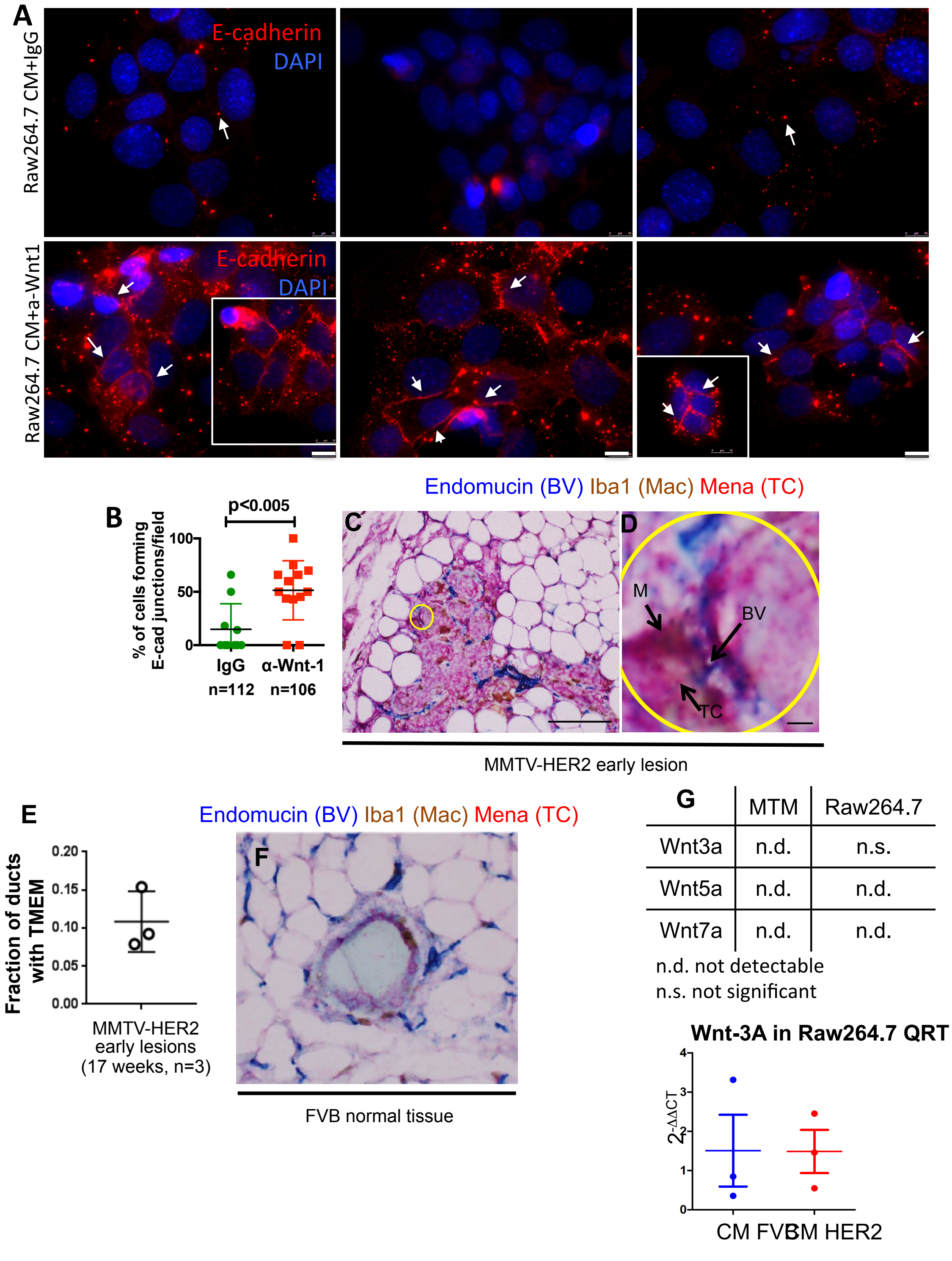

	MTM	Raw264.7
Wnt3a	n.d.	n.s.
Wnt5a	n.d.	n.d.
Wnt7a	n.d.	n.d.

Supplementary Figure 1. Macrophage secreted Wnt1-induced loss of E-cadherin junctions and TMEM formation in early lesions. a: IF detection of E-cadherin junctions in Comma-1D cells cultured for 24 hrs in the presence of conditioned media from RAW264.7 monocytic cells treated with an IgG control or with an antibody specific to mouse Wnt1 to deplete non-specific or Wnt1 proteins. Arrow point to globular E-cadherin not engaged in junctions in the top row and junctional E-cadherin in the bottom row. Bars 10 *mm* **b:** quantification of the results in A showing the percent of DAPI+ cells forming E-cadherin junctions. n, number of scored cells. P-value calculated with Mann-Whitney test at 95% confidence. Detection of TMEM in MMTV-HER2 early lesions (**c**) and the yellow circle area showing an example of TMEM is magnified in **d**. Endothelial cells (BV) are labeled by endomucin blue, early cancer cells by MENA staining (TC) cells in pink/red and macrophages by IBA1 staining in brown (Mac). Arrows in D points to the indicated cell types Mac: M. scale bars: c 100 *µm*, d 10 *µm*. **e**: quantification of the frequency of TMEM/ early lesion ductal structure. Note that the frequency of 10% is in range with the detection if intraepithelial macrophages in Figure 1J in 14 weeks early lesions.

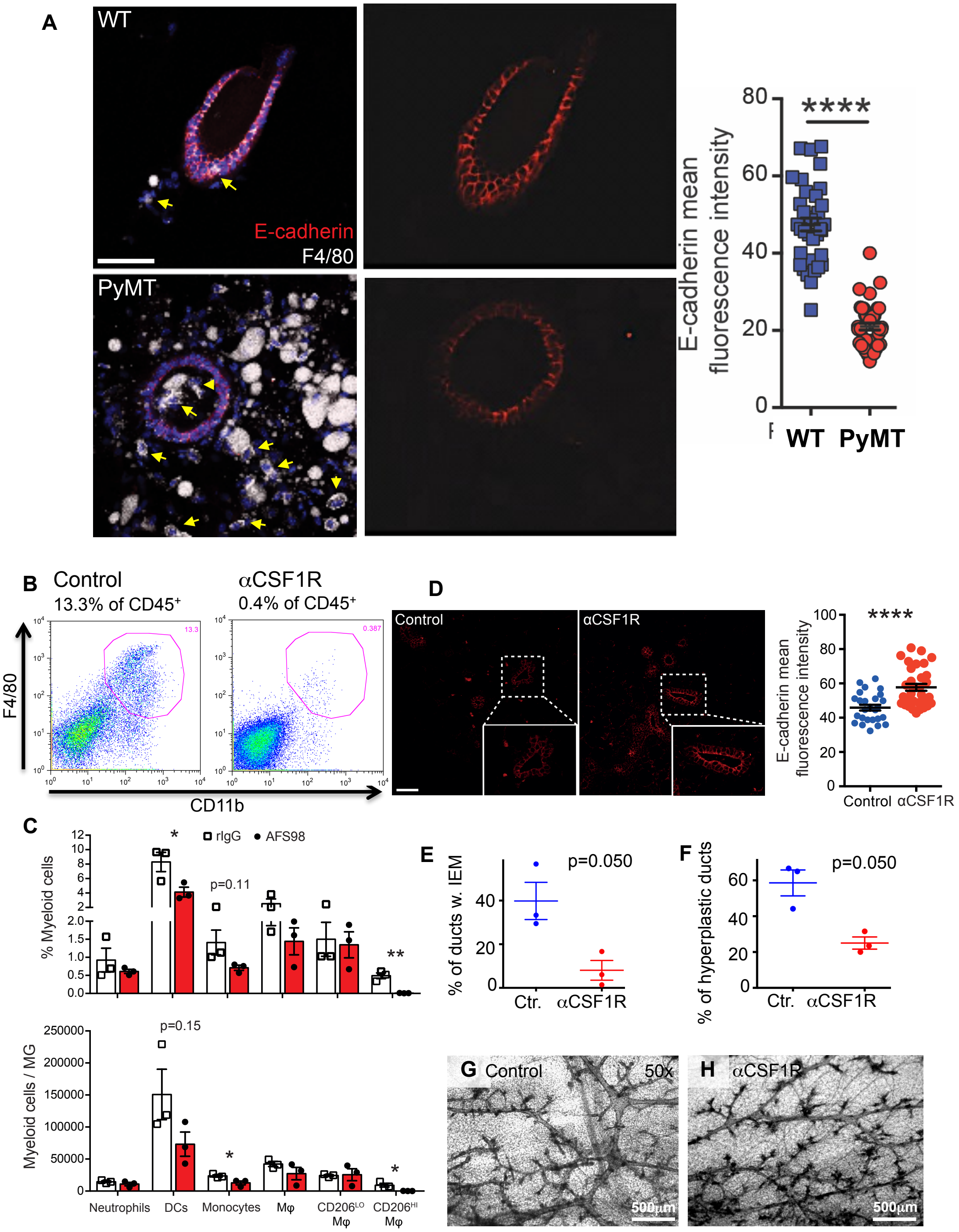

Supplementary Figure 2. E-cadherin junctions in early MMTV-PyMT lesions and effect of macrophage depletion on early lesion immune and epithelial profile. a: Immunofluorescence of wt (top) and MMTV-PyMT early lessions (bottom, 11 weeks old) showing macrophages (F4/80, white) and E-cadherin (red). Yellow arrows point to macrophages. Scale bar is 50 *µm*. Quantification of E-cadherin mean fluorescence pixel intensity is shown in wt and MMTV-PyMT on the graph on the right. N=26-32 ducts. p value<0.001 calculated with Mann-Whitney test at 95% confidence. **b**:_FACS measurement of mammary gland macrophage depletion (F4/80+/CD11b+) after two weeks of treatment with a CSF1R antibody compared to vehicle control (pre-malignant MMTV-HER2 20wks at start of treatment, 3 mice per group). **c:** Flow cytometry quantification of myeloid populations among CD45+ leukocytes in the mammary gland of wt rIgG (white bars) and AFS98-treated (red bars) of MMTV-Her2 females. N=3 females per group; * p≤0.05 paired t test**.** Plots represent mean values ± SEM. **d:** Representative immunofluorescence images showing E-cadherin signal for depletion experiment shown in C. Scale bar: 50 *µm*. Quantification of mean fluorescence intensity per duct is shown on the right graph. ****p≤ 0.0001 calculated by Mann-Whitney. **e:** Percentage of ducts with intra-epithelial macrophages (IEM; **e**) and hyperplastic ducts (**f**) was quantified in mammary gland sections (3 mice per group) after two weeks of CSF1R antibody or vehicle treatment shown in b. **g-h:** Whole mount staining of mammary glands after two weeks of vehicle control (**g**). and CSF1R antibody (**h**) treatment. Scale bars are shown for each panel.

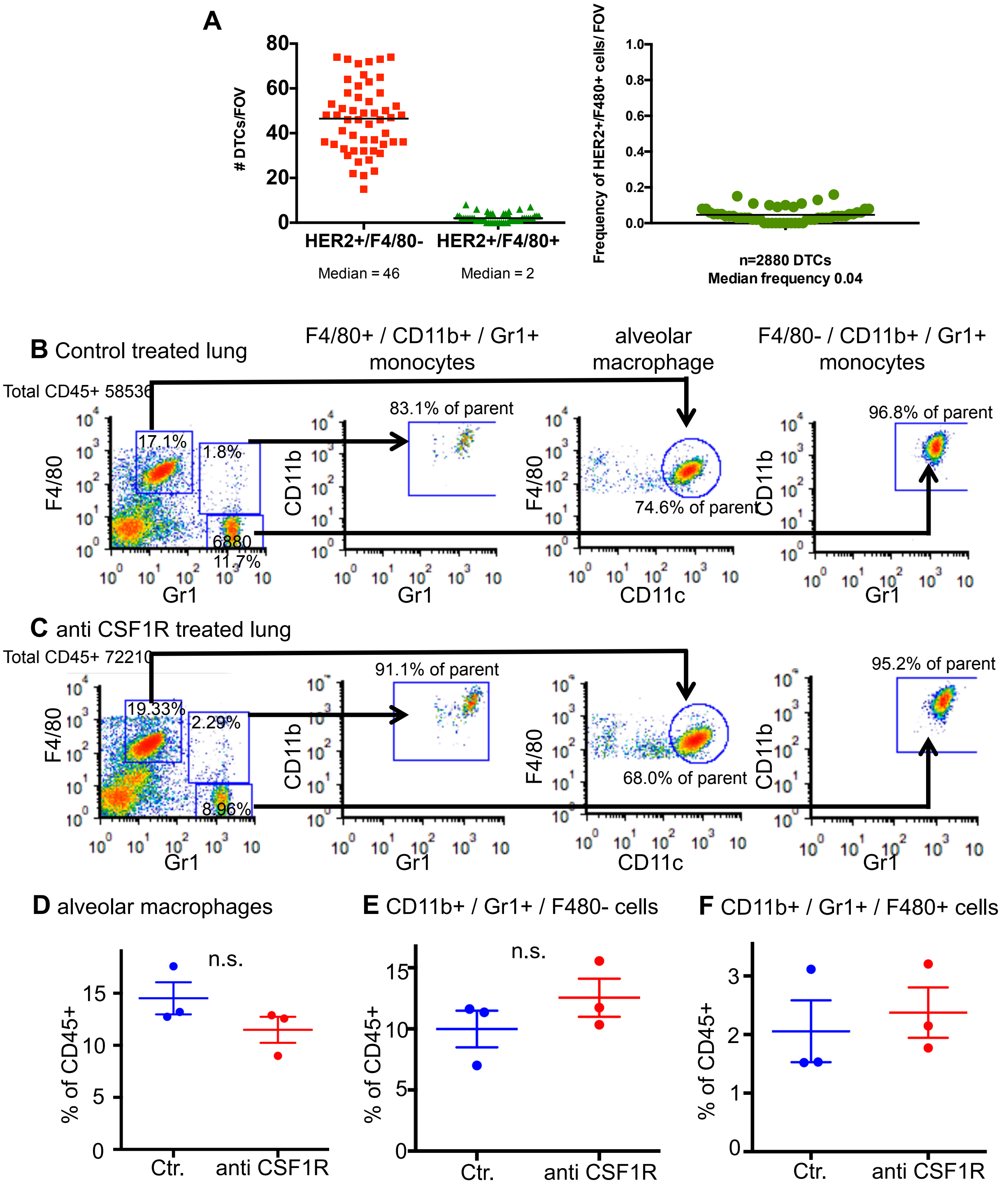

Supplementary Figure 3. Controls for macrophage engulfment of early HER2+ DCCs and gating strategy for macrophage subtype analysis. a: Quantification of HER2+/F4/80- and HER2+/F4/80+ cells in lung sections at 40X magnification; 60 fields were scored. Note that majority of cells (96%) when HER2+ are F4/80-. **b, c:** Flow cytometry lung gating strategy were CD45+ viable cells were identified as F4/80+/Gr1+/CD11b+ and F4/80-/Gr1+/CD11b+ monocytes and F4/80+/Gr1-/CD11c^{hi} alveolar macrophages. Quantification of alveolar macrophage (**d**) and monocyte populations (**e,f**) in lungs of MMTV-HER2 mice (3 per group) that had been treated with a CSF1R antibody or vehicle control during pre-malignant stages and were then allowed to progress to over tumor stages (Fig.4). Statistical testing: Mann-Whitney with 95% confidence.

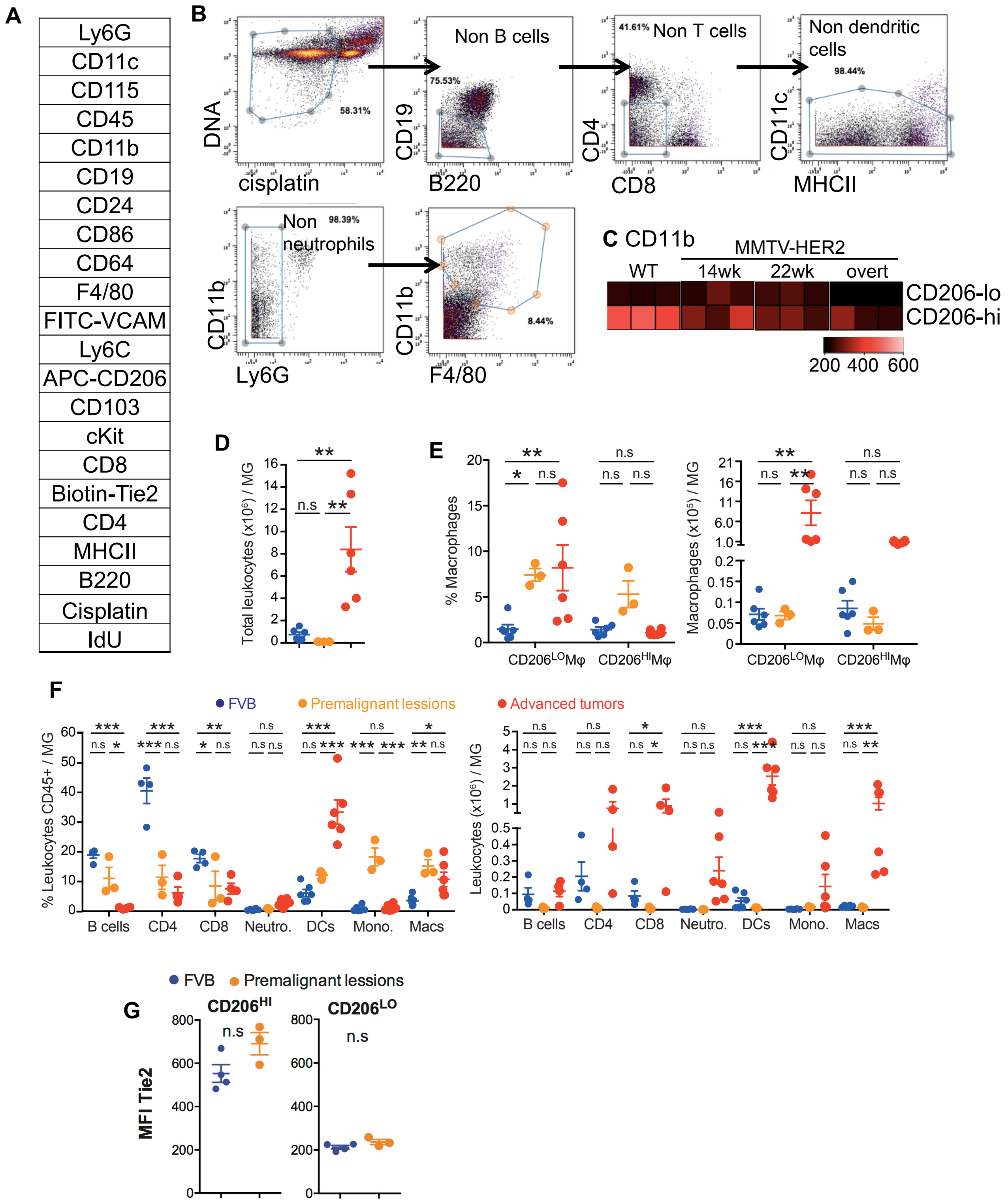

Supplementary Figure 4. Immune profiling of early lesions using CyTOF and multi-color FACS. a: List of markers analyzed by mass cytometry (CyTOF). **b**: Gating scheme to create viSNE plots of macrophages and monocytes. DNA staining and Cisplatin was used to identify viable cells. **c**: Heatplot of CD11b in the three myelo-monocytic populations identified by visne plot (Fig.2A). N=3 animals per group; independent experiments: N=2. **d:** CD45+ leuckocytes in wt (blue), early lessions (yellow) and overt tumors (red) in mammary glands of MMTV-Her2 females. N=3-5 mice per group. n.s, not significant; ** p≤ p0.01. Plots represent mean values ± SEM. **e:** Frequency (left) and absolute number (right) of macrophages subsets CD206^{LO} and CD206^{HI} among CD45+ leukocytes. Mφ**,** macrophage. Plots represent mean values ± SEM. **f:** Frequency of lymphoid and myeloid cells among CD45+ leukocytes (left) and absolute numbers (right) in the mammary glands (MG) shown in E. Neutro, neutrophils; DCs, dendritic cells; mono, monocytes; macs, macrophages. n.s., not significant; * p≤ p0.05; ** p≤ p0.01; *** p≤ p0.001. Statistical test: 2 way ANOVA. Plots represent mean values ± SEM. **g:** Mean fluorescence intensity of Tie2 for CD206^{HI} and CD206^{LO} macrophage subsets in wt and MMTV-Her2 females early lesions (20-24wks, 3-5 mice per group) as measured by multi-color FACS.

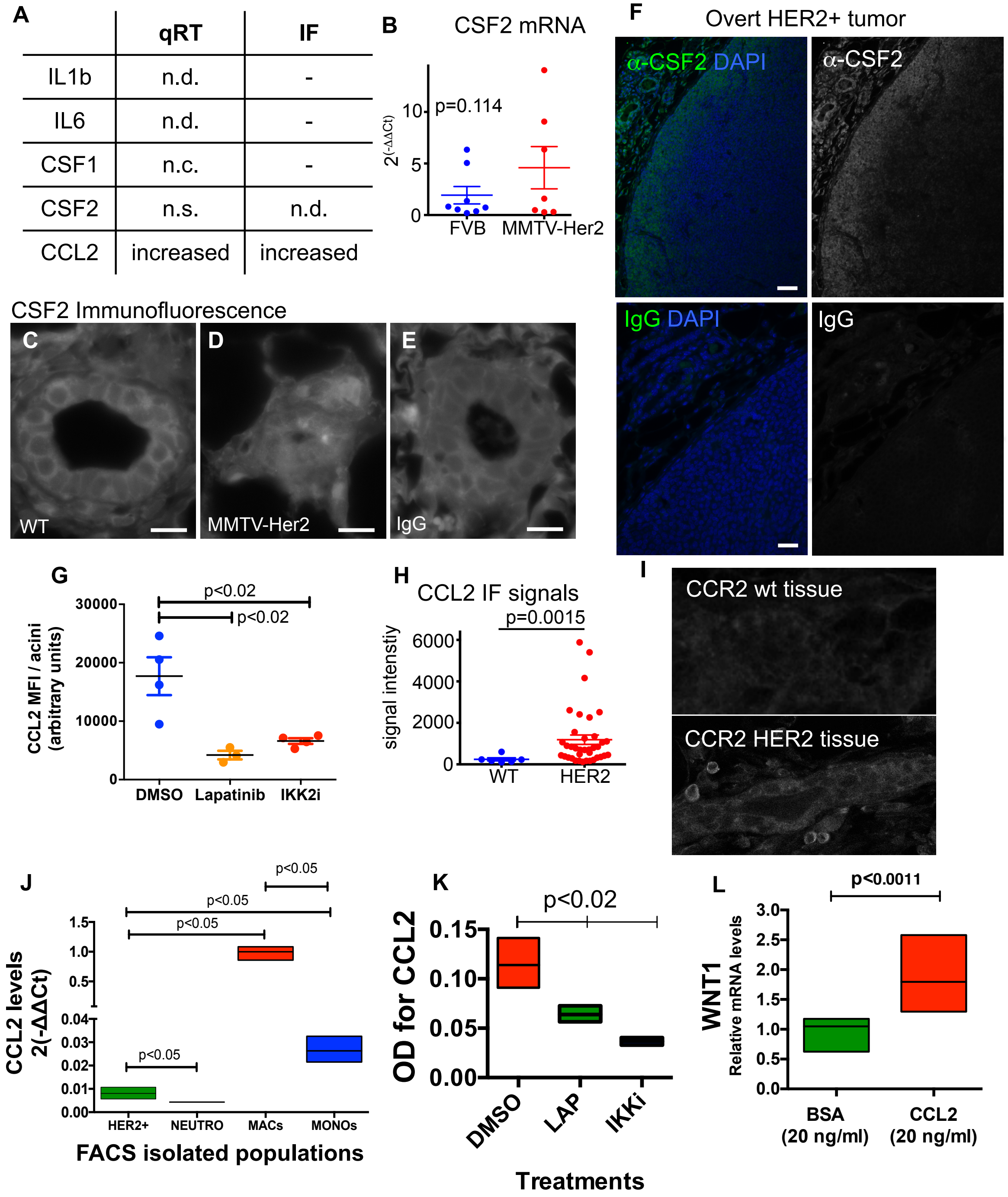

Supplementary Figure 5. Analysis of macrophage recruiting cytokines in early lesions. a: Results for qRT-PCR expression and immunofluorescent analysis results of selected cytokines in mammospheres derived from 20wk old MMTV-Her2 mammary glands that were not detectable (n.d.), did not change (n.c.) or did not chance significantly (n.s.) or were increased in relation to expression levels in FVB wild type mammary glands. **b**: qRT-PCR expression analysis of CSF2 expression in mammospheres derived from either FVB wild type or 20wk old MMTV-Her2 pre-malignant mice. Technical replicates of three mice each are shown. Student's t-test was used for statistical analysis.. **c-e**: Immunofluorescent staining (negative) of FVB wild type (**c**) and 22wk old MMTV-Her2 mammary glands (**d**) against CSF2 or with IgG control (**e**). Bars 25 *mm*. **f:** detection of CSF2 in overt MMTV-HER2 tumors (26-30 wks) supports that CSF2 is not detectable at protein level in early lesions. Bars 25 *mm*. **g:** Quantification of the mean fluorescence intensity for CCL2 staining in independent acini in Fig 6 e-g; n=2 independent experiment and multiple acini per group. **h:** quantification of the HER2 signal in Fig.6C, middle panel n=2 experiments. **i:** signal for CCR2 staining in the same tissues in Fig6c showing the signal in the whole fields and not only in selected areas exemplified in Fig6c. Bars 25 *mm*. **j:** qRT-PCR for CCL2 in the sorted cell populations indicated: HER2+, HER2+ early cancer cells; Neutro, neutrophils in the early lesion mammary glands; MACs, macrophages in the early lesion mammary glands; MONOs, monocytes in the early lesion mammary glands. **k:** quantification by ELISA of CCL2 secreted to the 24 hrs conditioned media of oncospheres produced by early cancer cells treated with control treatment (DMSO), lapatinib (LAP) or an IKK inhibitor (IKKi). **l:** qRTPCR for Wnt1 mRNA detected in RAW2647.7 monocytic cells treated with 20 *ng per ml* CCL2 for 24 hrs. All p values in all figure panels were calculated with Mann-Whitney test at 95% confidence.

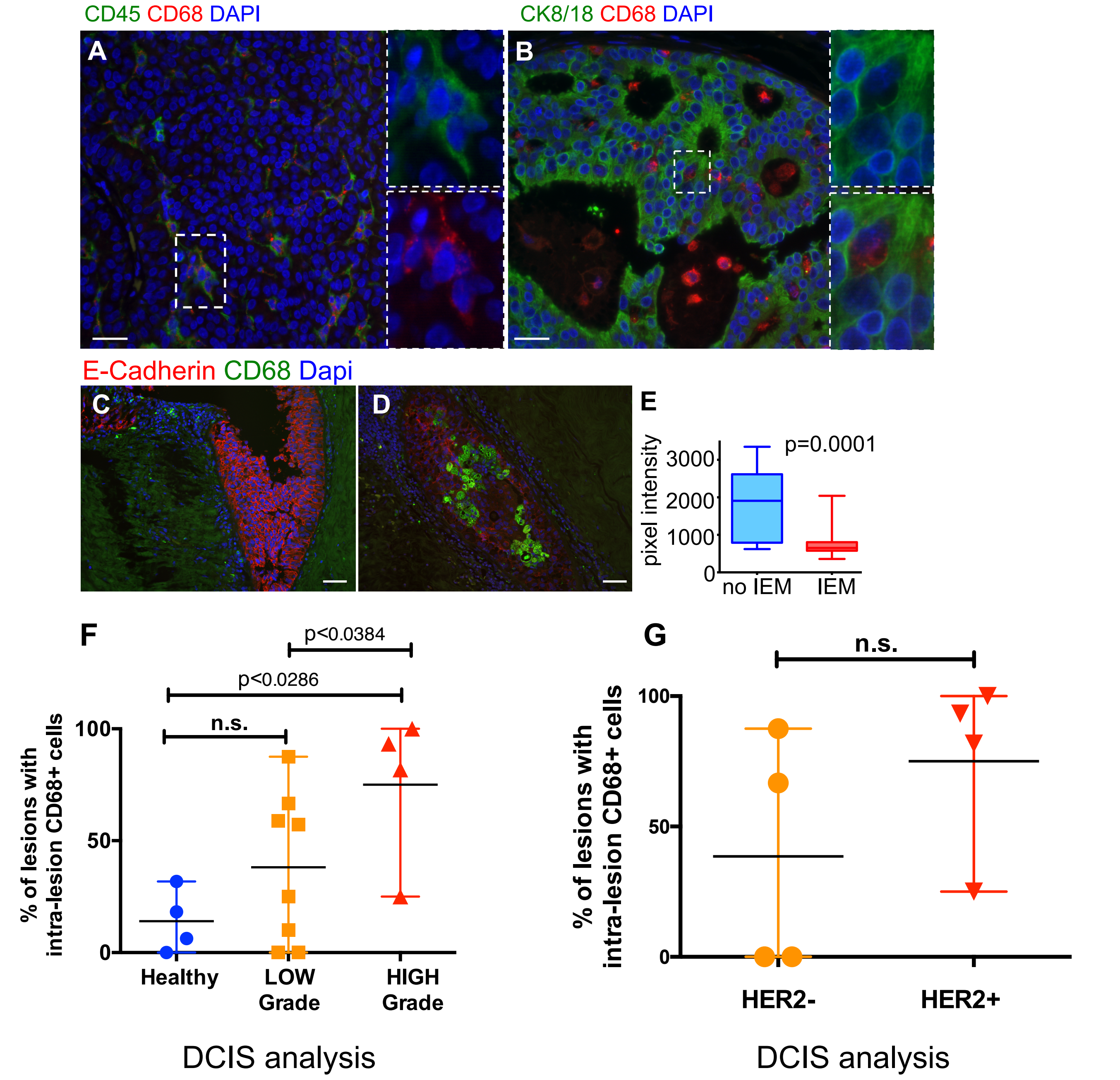

Supplementary Figure 6. Analysis of human DCIS samples. Human DCIS sections were stained against CD68 and CD45 (**a**) and CD68 and cytokeratin 8/18 (**b**). **b,c**: Two individual ducts in one DCIS lesion of the same patient with either high E-Cadherin and low intra-epithelial macrophage (IEM) levels (**c**) or low E-Cadherin levels and high IEM levels (**d**) within the same patient. **e**: Quantification of E-Cadherin signal intensity in the region of cell junctions within ducts of the same patients with either high or low numbers of IEMs**f:** Quantification of the frequency of macrophage infiltration in healthy adjacent and DCIS lesions separated by high and low grade subtype. Each point is a patient sample. **g**: Quantification of the frequency of macrophage infiltration in healthy adjacent and DCIS lesions separated by HER2 status. Each point is a patient sample. All p values in all figure panels were calculated with Mann-Whitney test at 95%confidence.

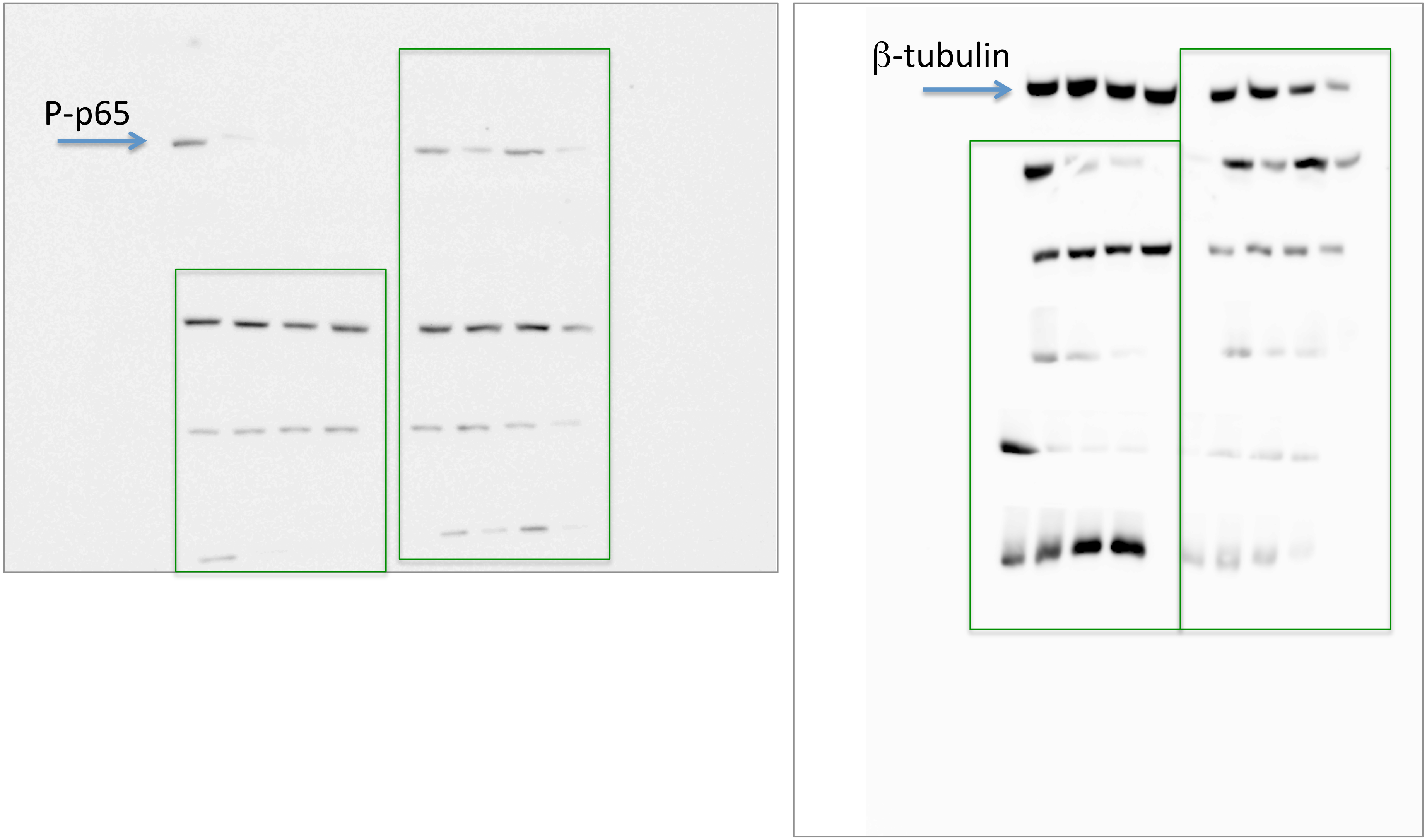

Supplementary Figure 7. The panels in this figure show the un-cropped blots for the P-p65 and β-tubulin detection blots shown in Fig6A. Arrows point to the bands in Fig6A and the green boxes demark unrelated experiments.

Supplementary Table 1

Animal information for Fig.1

Figure	Experiment	Mouse strain	Age (weeks)	Mouse gender	Number of mice
Fig.1a)	H&E staining mammary gland	FVB	20	female	3-6
Fig.1b)	H&E staining mammary gland	MMTV-HER2	22	female	4-6
Fig.1c)	H&E staining tumor	MMTV-HER2	26-30	female	4-6
Fig.1d, 1g, 1j	IF staining and quantification	FVB	20	female	4
Fig.1e, 1h, 1j)	IF staining and quantification	MMTV-HER2	14	female	3
Fig.1f, 1i, 1j)	IF staining and quantification	MMTV-HER2	22	female	5

Supplementary Table 2

Animal information for Fig.2

Figure	Experiment	Mouse strain	Age (weeks)	Mouse gender	Number of mice
Fig.2a-c)	IF staining and quantification	MMTV-HER2	20	female	4
Fig.2d)	Quantitative PCR of mammary glands	FVB	20	female	4
		MMTV-HER2	20	female	7
Fig.2e-g)	IF staining and quantification	MMTV-HER2	24	female	3
Fig.2k,l)	Conditioned medium from primary mammospheres	FVB	20-22	female	2-3

Supplementary Table 3

Animal information for Fig.3

Figure	Experiment	Mouse strain	Age (weeks)	Mouse gender	Number of mice
Fig.3b,c)	H&E staining mammary gland	MMTV-HER2	22	female	6-7
Fig.3d)	Quantitative PCR of mammary glands	MMTV-HER2	22	female	6-7
Fig.3e-g)	IF staining and quantification	MMTV-HER2	22	female	3
Fig.3h)	Circulating early cancer cells	MMTV-HER2	22	female	7
Fig.3i-k)	IF staining and quantification	MMTV-HER2	22	female	6-7

Supplementary Table 4

Animal information for Fig.4

Figure	Experiment	Mouse strain	Age (weeks)	Mouse gender	Number of mice
Fig.4b)	Time until tumor onset	MMTV-HER2	23-38	female	9
Fig.4c)	Time until tumors were overt	MMTV-HER2	26-43	female	6-9
Fig.4d-f)	IF staining and quantification	MMTV-HER2	26-43	female	3-4
Fig.4g-i)	IF staining and quantification	MMTV-HER2	26-43	female	3-4
Fig.4j-l)	IF staining and quantification	MMTV-HER2	26-43	female	4-6

Supplementary Table 5

Animal information for Fig.5

Figure	Experiment	Mouse strain	Age (weeks)	Mouse gender	Number of mice
Fig.5a-f, 5i-j)	CyTOF	FVB	20-24	female	5
		MMTV-HER2	14	female	5
		MMTV-HER2	22	female	5
Fig.5g,h, 5i-j)		MMTV-HER2	26-30	female	5
Fig.5k-n)	IF staining and quantification	FVB	20-24	female	3
		MMTV-HER2	24	female	3
		MMTV-HER2	26-30	female	3

Supplementary Table 6

Animal information for Fig.6

Figure	Experiment	Mouse strain	Age (weeks)	Mouse gender	Number of mice
Fig.6a)	Western Blot with primary mammospheres	MMTV-HER2	20	female	3
Fig.6b)	Quantitative PCR of primary mammospheres	FVB	20-24	female	3
		MMTV-HER2	22	female	3
Fig.6c)	IF staining and quantification	FVB	20-24	female	3
		MMTV-HER2	22	female	3
Fig.6d)	OD for CCL2 via Elisa	FVB	20-24	female	2
		MMTV-HER2			
Fig.6e-g)	IF staining and quantification	MMTV-HER2	20-24	female	2

Fig.6h-j)	IF staining and quantification	MMTV-HER2	20	female	2
Fig.6k-m)	IF staining and quantification	MMTV-HER2	22	female	6-8
Fig.6n,o)	IF staining and quantification	MMTV-HER2	22	female	5

Supplementary Table 7

Animal information supplementary Figures

Figure	Experiment	Mouse strain	Age (weeks)	Mouse gender	Number of mice
Sup Fig.1c-e)	IHC and quantification	MMTV-HER2	17	female	3
Sup Fig.2a)	IF and quantification	WT	11	female	1
Sup Fig.2a)	IF and quantification	MMTV-PyMT	11	female	1
Sup Fig.2b)	Flow cytometry	MMTV-HER2	22	female	3
Sup Fig.2c,d)	Flow cytometry	MMTV-HER2	20-24	female	3
Sup Fig.2e,f)	IF and quantification	MMTV-HER2	22	female	3
Sup Fig.2g,h)	Whole mount staining	MMTV-HER2	22	female	3
Sup Fig.3a)	IF and quantification	MMTV-HER2		female	2
Sup Fig.3b-f)	Flow cytometry	MMTV-HER2	26-43	female	3
Sup Fig.4a-c	CyTOF	FVB	20-24	female	5
		MMTV-HER2	20-24	female	5
Sup Fig.4d-g)	Flow cytometry	MMTV-HER2	20-24	female	3-5
Sup Fig.5b)	Quantitative PCR of mammospheres	FVB, MMTV-HER2	20	female	3
Sup Fig.5c-e)	Immunofluorescence	FVB, MMTV-HER2	22	female	3
Sup Fig.5f)	Immunofluorescence	MMTV-HER2	26-30	female	3
Sup Fig.5g)	IF staining and quantification	MMTV-HER2	20-24	female	2
Sup Fig.5h,i)	IF staining and quantification	FVB, MMTV-HER2	20-24	female	3
Sup Fig 5J	Qpcr ccl2	MMTV-HER2	18	female	4

Sup Fig.5K)	ELISA	MMTV-HER2		female	2
Sup Fig.5h,i)	IF staining and quantification	FVB, MMTV-HER2	20-24	female	3
